# Associations between green/blue spaces and mental health across 18 countries

**DOI:** 10.1038/s41598-021-87675-0

**Published:** 2021-04-26

**Authors:** Mathew P. White, Lewis R. Elliott, James Grellier, Theo Economou, Simon Bell, Gregory N. Bratman, Marta Cirach, Mireia Gascon, Maria L. Lima, Mare Lõhmus, Mark Nieuwenhuijsen, Ann Ojala, Anne Roiko, P. Wesley Schultz, Matilda van den Bosch, Lora E. Fleming

**Affiliations:** 1 Cognitive Science HUB, University of Vienna, Liebbigasse 5, 1110 Vienna, Austria; 2 European Centre for Environment and Human Health, University of Exeter Medical School, Exeter, UK; 3 Institute of Psychology, Jagiellonian University, Krakow, Poland; 4 College of Engineering, Mathematics, and Physical Sciences, University of Exeter, Exeter, UK; 5 Estonian University of Life Sciences, Tartu, Estonia; 6 School of Environmental and Forest Sciences, College of the Environment, University of Washington, Washington, USA; 7 ISGlobal, Barcelona, Spain; 8 Universitat Pompeu Fabra (UPF), Barcelona, Spain; 9 CIBER Epidemiología y Salud Pública (CIBERESP), Madrid, Spain; 10 Department of Social and Organizational Psychology, ISCTE – University Institute of Lisbon, Lisbon, Portugal; 11 Institute of Environmental Medicine, Karolinska Institute, Solna, Sweden; 12 Natural Resources Institute Finland (Luke), Helsinki, Finland; 13 School of Medicine, Griffith University, Brisbane, Australia; 14 Department of Psychology, California State University San Marcos, San Marcos, USA; 15 School of Population and Public Health, University of British Columbia, Vancouver, Canada; 16 Department of Forest and Conservation Sciences, University of British Columbia, Vancouver, Canada

**Keywords:** Psychology, Environmental social sciences

## Abstract

Living near, recreating in, and feeling psychologically connected to, the natural world are all associated with better mental health, but many exposure-related questions remain. Using data from an 18-country survey (*n* = 16,307) we explored associations between multiple measures of mental health (positive well-being, mental distress, depression/anxiety medication use) and: (a) exposures (residential/recreational visits) to different natural settings (green/inland-blue/coastal-blue spaces); and (b) nature connectedness, across season and country. People who lived in greener/coastal neighbourhoods reported higher positive well-being, but this association largely disappeared when recreational visits were controlled for. Frequency of recreational visits to green, inland-blue, and coastal-blue spaces in the last 4 weeks were all positively associated with positive well-being and negatively associated with mental distress. Associations with green space visits were relatively consistent across seasons and countries but associations with blue space visits showed greater heterogeneity. Nature connectedness was also positively associated with positive well-being and negatively associated with mental distress and was, along with green space visits, associated with a lower likelihood of using medication for depression. By contrast inland-blue space visits were associated with a greater likelihood of using anxiety medication. Results highlight the benefits of multi-exposure, multi-response, multi-country studies in exploring complexity in nature-health associations.

## Introduction

Poor mental health is the leading cause of disease burden in high-income countries^1^. This may, at least in part, be a consequence of rapid urbanisation^2, 3^ and a growing disconnection from the natural world^4, 5^. A growing body of research suggests that living near and/or maintaining regular contact with nature is beneficial for a range of health and well-being outcomes^6–8^, but several issues remain outstanding^9^.

First, there is a lack of clarity about the relative importance of merely living near nature, variously referred to as residential proximity, neighbourhood exposure or indirect contact^10^, compared to more direct interactions including deliberate engagement through recreational visits^11^. Although some benefits to mental health and well-being may result from mere neighbourhood exposure, e.g. reduced noise and air pollution and lower temperatures, others are thought to derive from voluntarily spending time in natural settings for relaxation, meeting others, and/or undertaking physical exercise^10, 12^. To date, the vast majority of studies have focused on residential proximity^13^ and although a positive association is sometimes reported with recreational visits^14, 15^, there is also evidence that many people rarely visit local nature^16^, while others travel, sometimes quite far, outside of their neighbourhood for exercise and nature-based recreation^17, 18^. Proximity is a far from perfect proxy for use.

Second, emerging evidence suggests that mental health may be non-linearly related to recreational exposure, with diminishing marginal returns beyond a certain threshold^19^. As with many other ‘goods’, it may be that the benefits of nature-based recreation become less pronounced with each additional visit. Greater clarity about the relative importance of residential exposure and recreational visits, as well as their potentially non-linear relationships, is critical in designing public health interventions that not only improve availability but also support the most appropriate levels of use, both locally and further afield.

Third, most research has operationalised nature in terms of ‘green space’ (e.g. parks, woodlands, street trees, vegetation cover) and under-explored the potential role of both inland-blue spaces (e.g. rivers, lakes)^20, 21^, and coastal-blue spaces (e.g. beaches, promenades)^22^, for mental health. Although green and blue spaces share many qualities (e.g. cooling effects, biodiversity), blue spaces also offer alternative recreational activities (e.g. swimming) and have additional features (e.g. unique soundscapes)^23–25^. It is only through examining both in tandem that we will get a clearer idea of their relative potential benefits for mental health.

Fourth, the field has used a wide range of mental health metrics, including indices of both positive and negative mental health^6, 8, 10, 12^. Rates of poor mental health tend to be lower among populations living in greener neighbourhoods^26–28^, and one-off nature walks have been shown to reduce symptoms of anxiety/depression in at-risk populations^29, 30^. However, there has been relatively little large-scale research exploring relationships between voluntary, recreational time in nature and indicators of mental health^11, 19, 31^. This is important because meta-analyses suggest that the benefits of direct nature exposure tend to have a larger effect on promoting positive emotions than reducing negative ones^32^, and thus it may be that indicators of positive mental health are more sensitive to recreational visits than negative ones. Again, this is best explored in studies that include multiple exposure metrics alongside multiple mental health outcomes.

Fifth, research suggests that psychological connectedness to the natural world, e.g. feeling part of nature or seeing beauty in natural things, is also positively associated with positive well-being^33^. Given that people high in nature connectedness also tend to report more recreational visits^34, 35^, any positive association between visits and well-being may be due to the underlying nature connectedness an individual has, rather than a product of the environment itself. To unpack this possibility, more research is needed to explore the simultaneous relationships between exposures, nature connectedness and mental health, so that their unique roles can be identified.

Finally, there may be important seasonal and societal/cultural differences in the way nature affects mental health^9, 10, 12^. For instance, most research using the Normalized Differential Vegetation Index (NDVI) as its measure of residential green space uses summer data, and applies it to health data for the whole year even though relationships may be different when leaf cover is lower in winter months^10^. Similarly, blue spaces may be better for mental health in summer/autumn when the water temperatures are higher^36^. Living near and spending time in green and blue space is also likely to be quite different, for instance, in southern European countries than northern European countries. Not only are temperatures and vegetation different, hours of daylight vary substantially across the year potentially affecting time outdoors^36, 37^.

The current research used a large international survey in an attempt to begin to address these issues. We collected data on both residential exposure, using satellite imagery of a 1000 m buffer around the home, and recreational visits, using self-reported visit frequency in the last four weeks. We also explored whether individuals had both inland-blue and coastal-blue space within 1000 m buffers of their home, and how often they had visited each type of blue space in the last 4 weeks. We collected measures of both positive and negative mental health. Following earlier studies in the field^38, 39^ we asked participants to complete the World Health Organisation’s 5-item index of *positive well-being*. The aggregate 100-point WHO-5 scale has the additional benefit that low scores (i.e. < 28) are indicative of being at risk of depression/anxiety^40, 41^, and are thus an indicator of *mental distress*. Additionally, we included two questions from the European Health Interview Survey that asked about recent use of doctor-prescribed *medication* for *depression* and *anxiety*^42^. To explore the role of nature connectedness, we included the Inclusion of Nature in Self (INS) scale^43, 44^. Finally, our survey was conducted at four times during a 12-month period, to explore seasonal effects, and across 18 countries/regions to explore generalisability across locations.

We investigated four hypotheses (H). H1: Greater residential exposure to green, inland-blue and coastal-blue spaces will be associated with (a) higher positive well-being, (b) lower probability of mental distress, and lower probability of medication use for (c) depression and (d) anxiety. H2: More frequent recreational visits to these three settings will show similar relations to those for residential exposure for the four outcomes. H3: The positive association between visits and mental health in H2 will be non-linear and show diminishing marginal returns. H4: Psychological connectedness to nature will be a significant independent predictor of mental health outcomes over and above residential exposure and recreational contact. Two more exploratory research questions (RQs) focused on the consistency of any overarching relationships found between nature exposure, connectedness and mental health across season (RQ1) and country (RQ2).

Hypotheses were tested using a series of linear mixed effects models for WHO-5 scores, and Bernoulli generalised linear mixed effects models for the binary outcomes of mental distress and medication use. Main models included: (a) residential exposure, (b) recreational visits, and (c) nature connectedness; (d) quadratic (squared) terms for visit frequency and connectedness to test for non-linearity; and controlled for potential covariates. Analyses were re-run using stratification on: (a) season; and (b) country, to explore RQs (see “Materials and methods” section for more details).

## Results

Descriptive data for key predictors are presented in Table 1 and data for all covariates in Supplementary Table S1. Table 2 presents core model summaries with full models including all covariates presented in Supplementary Tables S2–S5. Due to space constraints in the text, descriptive data and covariates are only discussed in Supplementary Materials, and the 95% Confidence Intervals for estimates are reported in Tables and Figures. In order to maintain model power for our more exploratory questions into seasonal and country variation we focused on the WHO-5 positive well-being scores, rather than the dichotomous indices of mental distress and medication use.

**Table 1:** The Ns, percentages (%), means (Ms), standard deviations (SDs), and correlations (r / r_pb_) for the four mental health outcomes as a function of residential exposure (Q = quartile), recreational visits and nature connectedness for the analytical sample (n = 16,302).

	n	%	M	SD	WHO-5		WHO-5 < 28		Depression Meds		Anxiety Meds	
					M/r	SD	N/r_pb_	%	N/r_pb_	%	N/r_pb_	%
**Residential exposure [within 1000 m]**												
Greenspace [Q1]	4103	25.17	1.36	1.87	58.79	21.55	381	9.29	354	8.63	366	8.92
Greenspace [Q2]	4098	25.14	19.79	9.35	59.73	21.57	352	8.59	362	8.83	389	9.49
Greenspace [Q3]	4071	24.97	62.11	14.44	61.29	21.53	333	8.18	330	8.11	374	9.19
Greenspace [Q4]	4030	24.72	96.85	4.18	60.86	22.17	352	8.73	405	10.05	416	10.32
Inland blue [no]	10,141	62.21	NA	NA	60.27	22.00	897	8.85	872	8.60	962	9.49
Inland blue [yes]	6161	37.79	NA	NA	59.98	21.25	521	8.46	579	9.40	583	9.46
Coastal blue [no]	14,507	88.99	NA	NA	60.04	21.77	1272	8.77	1330	9.17	1410	9.72
Coastal blue [yes]	1795	11.01	NA	NA	61.11	21.35	146	8.13	121	6.74	135	7.52
**Recreational visits [last 4 weeks]**												
Green	NA	NA	12.34	12.85	0.26***	NA	− 0.12***	NA	− 0.01	NA	0.03***	NA
Inland blue	NA	NA	6.08	8.95	0.19***	NA	− 0.08***	NA	0.03*	NA	0.06***	NA
Coastal blue	NA	NA	5.34	10.17	0.18***	NA	− 0.07***	NA	0.00	NA	0.05***	NA
**Nature connectedness**												
INS	NA	NA	4.14	1.65	0.24***	NA	− 0.11***	NA	− 0.04***	NA	− 0.03***	NA

*r* Pearson’s correlation, *r*_*pb*_ point bi-serial correlation (due to binary outcome); *INS =* inclusion of nature in self scale.

***p < 0.001; see Supplementary Table S1 for details of all covariates.

**Table 2:** Mental health as a function of residential exposure, recreational visits and nature connectedness controlling for socio-demographics, season and country.

Predictors	WHO-5 scale (0–100)		WHO-5 distress (< 28)		Depression medication use		Anxiety medication use	
	Estimates	95% CIs	Odds ratios	95% CIs	Odds ratios	95% CIs	Odds ratios	95% CIs
(Intercept)	48.15***	46.06, 50.24	0.19***	0.14, 0.27	0.06***	0.04, 0.09	0.06***	0.04, 0.09
**Residential exposure [within 1000 m]**								
Greenspace [Q2 vs. Q1]	0.46	− 0.38, 1.30	0.94	0.80, 1.11	0.99	0.83, 1.19	1.15	0.97, 1.36
Greenspace [Q3 vs. Q1]	1.01*	0.15, 1.87	0.93	0.78, 1.10	0.85	0.71, 1.03	1.03	0.86, 1.24
Greenspace [Q4 vs. Q1]	0.37	− 0.51, 1.25	1.02	0.86, 1.21	0.99	0.82, 1.19	1.05	0.88, 1.25
Inland blue [Yes vs. No]	− 0.08	− 0.74, 0.58	0.94	0.82, 1.07	0.96	0.83, 1.10	1.00	0.87, 1.14
Coastal blue [Yes vs. No]	0.74	− 0.31, 1.79	1.01	0.81, 1.25	0.90	0.71, 1.15	0.82	0.65, 1.03
**Recreational visits [last 4 weeks]**								
Green	0.26***	0.22, 0.30	0.97***	0.96, 0.98	0.99*	0.98, 1.00	1.00	0.99, 1.01
Green^2^	− 0.00*	− 0.00, − 0.00	1.00*	1.00, 1.00	1.00*	1.00, 1.00	1.00	1.00, 1.00
Inland blue	0.12***	0.05, 0.19	0.97***	0.96, 0.99	1.01	0.99, 1.02	1.02*	1.00, 1.03
Inland blue^2^	− 0.00	− 0.00, 0.00	1.00**	1.00, 1.00	1.00	1.00, 1.00	1.00	1.00, 1.00
Coastal blue	0.19***	0.12, 0.25	0.97***	0.96, 0.99	0.99	0.98, 1.01	1.01	1.00, 1.02
Coastal blue^2^	− 0.00	− 0.00, 0.00	1.00*	1.00, 1.00	1.00	1.00, 1.00	1.00	1.00, 1.00
**Nature connectedness**								
INS	2.35***	1.45, 3.25	0.62***	0.52, 0.72	0.83*	0.70, 1.00	0.96	0.81, 1.14
INS^2^	− 0.09	− 0.20, 0.01	1.05***	1.03, 1.07	1.02	1.00, 1.04	1.00	0.98, 1.02
**Random effects**								
18 country intercept variance^a^	6.34		0.07		0.18		0.13	
Observations	16,302		16,302		16,302		16,302	
Marginal R^2^/Conditional R^2^	0.216/0.230		0.235/0.250		0.315/0.351		0.240/0.269	

Analyses used survey weights.

*INS* inclusion of nature in self scale.

^a^Variance of country-level intercepts from the random effects component of the model; Marginal R^2^ includes only fixed effects and Conditional R^2^ includes the random country effect, R^2^ for binary outcomes = Nakawaga Pseudo R^2^. Models control for sex, age, household income, employment status, education, long-term illness/disability, marital status, number of adults and children in household, dog and car ownership, weekly physical activity, season of data collection, and use of the alternative depression/anxiety medication for medication models only; full models in Supplementary Tables S2 and S3.

*p < 0.05, **p < 0.01, ***p < 0.001.

### Residential exposure (H1)

There was limited support for Hypothesis 1. The only significant association between residential exposure and mental health was for the WHO-5 scores for the 3rd versus 1st quartile of greenspace (*β* = 1.01; *p* < 0.05; Table 2). This was partly due to the inclusion of visit frequency in the main model. Without visit frequency, but with socio-demographic controls (Supplementary Table S2), there were *also* positive associations between living in quartile 4 (*vs*. quartile 1) of greenspace (*β* = 1.78, *p* < 0.001) and living within 1000 m of the coast (*β* = 1.98; *p* < 0.001). There were no associations between residential exposure and mental distress or depression/anxiety medication use in models including or excluding visit frequency (Supplementary Tables S2, S3).

### Recreational visits (H2 and H3)

Supporting Hypothesis 2, the linear terms for visit frequencies were significantly positively associated with WHO-5 scores: green space (*β* = 0.26; *p* < 0.001); inland-blue space (*β* = 0.12; *p* < 0.001); coastal-blue space (*β* = 0.19; *p* < 0.001), and negatively associated with the likelihood of mental distress (WHO-5 < 28; all three *ORs* = 0.97; *p* < 0.001). The likelihood of using depression medication was also negatively associated with green space visit frequency (*OR* = 0.99, *p* < 0.05). In contrast, the likelihood of using anxiety medication was *positively* associated with inland-blue space visits (*ORs* = 1.02; *p* < 0.05).

**Figure 1:**
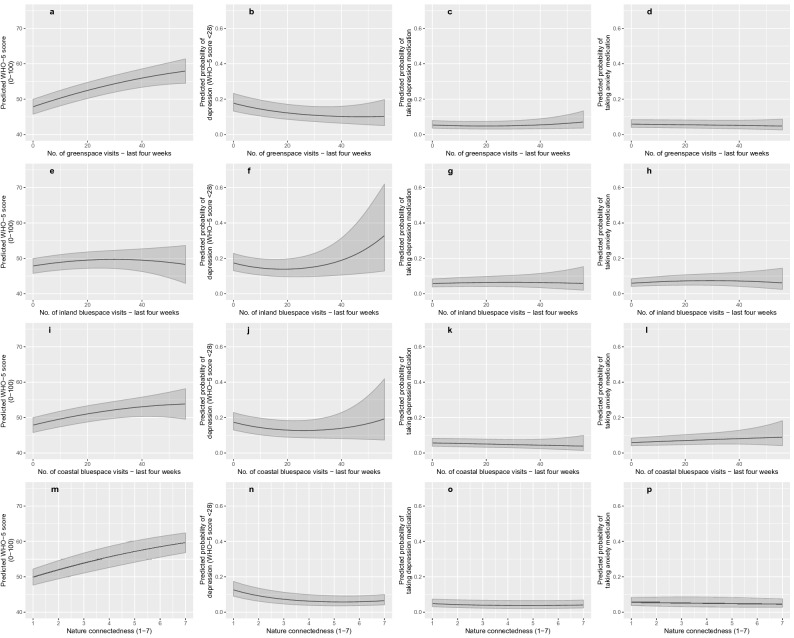
Relationships between: (1) Green space visits in last 4 weeks, (2) Inland-blue space visits in last 4 weeks, (3) Coastal-blue space visits in last 4 weeks, and (4) nature connectedness (1–7); and positive well-being (0–100; **a,e,l,m**), risk of mental distress (0–1; **b,f,j,n**), use of depression medication (0–1; **c,g,k,o**), and use of anxiety medication (0–1; **d,h,l,p**), averaged across 18 countries (n = 16,302). Plots are based on predicted values from linear and logistic mixed effects regression models including linear and quadratic terms (with 95% Confidence Intervals) for visit frequency and connectedness controlling for residential exposure, visit frequencies to alternative locations, connectedness (**a**–**l** only), age, gender, employment status, relationship status, household income, longstanding-illness, education level, household composition, dog ownership, car ownership, physical activity, season (sample wave), and country (as a random effect). Depression models also control for anxiety medication use and vice versa. Visit frequency was capped at n = 56 (i.e. two visits per day over 4 weeks). Covariates are held constant at their reference categories, or at their means for continuous predictors.

Partly supporting Hypothesis 3, there were also significant quadratic terms, indicative of non-linear diminishing marginal returns, for: (a) green space and inland-blue space visits and positive well-being (WHO-5); (b) all three visit types and mental distress (WHO-5 < 28); and (c) green space visits and depression medication use. However, because the estimates are based on only one extra visit per 4 weeks, the odds ratios are only visibly different from a null result at the third decimal. To aid interpretation, Fig. 1 plots the combined effects of the linear and quadratic terms for each visit type, for each outcome (panels a–l). Taking panel (a) as an example, the linear relationship between green space visits and WHO-5 is reflected in the positive upward slope, and the quadratic effect is reflected in the rate of increase getting gradually smaller and the curve beginning to flatten out. The wider confidence intervals to the right reflect fewer people visiting green spaces more than 40 times in the last four weeks and the curve ends at 56 visits due to our capping procedure at a maximum of two visits per day (see “Methods” section). The opposite effect occurs for measures of mental distress, e.g. panel (b) shows a decreased probability of reporting a WHO-5 score < 28 with each additional green space visit, but this decrease gets progressively smaller as the number of visits increases. The large confidence intervals for high levels of inland visits were due to the small number of people visiting these spaces > 40 times in the last four weeks.

### Nature connectedness (H4)

Supporting Hypothesis 4, nature connectedness was independently: (a) positively associated with positive well-being (*β* = 2.35, *p* < 0.001); (b) negatively associated with mental distress (*OR* = 0.62; *p* < 0.001), with diminishing marginal returns reflected in a significant quadratic term (*OR* = 1.05; *p* < 0.001); and (c) negatively associated with depression medication use (*OR* = 0.83, *p* < 0.05). These relationships are shown in panels m–p in Fig. 1. Note that the larger coefficients for connectedness are partly a reflection of the fact this was a seven-point scale (compared to the 0–56 scale for visits).

### Seasonality (RQ1)

**Figure 2:**
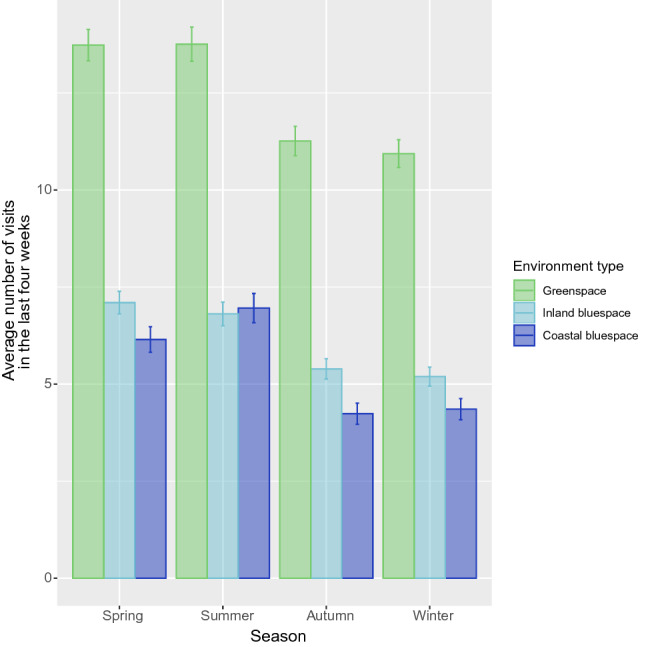
Average number of visits to green spaces, inland-blue spaces and coastal-blue spaces as a function of season across the whole sample (n = 16,302). Error bars represent 95% confidence intervals.

Figure 2 presents visit frequency for the last 4 weeks as a function of season. Despite the drop of approximately two visits in all three settings in autumn/winter, compared to spring/summer, visits to inland-blue and coastal-blue both remained at an average of just above 4 (i.e. once a week). The stratified results predicting positive well-being for each season are presented in Supplementary Table S4. Residential greenspace was only significantly associated with positive well-being for Q3 versus Q1 in spring (*β* = 1.78), and there continued to be no significant associations with either residential inland- or coastal- blue space in any season. In terms of visits, each additional green space visit was associated with significantly greater WHO-5 scores across all four seasons (spring *β* = 0.24, summer *β* = 0.22, autumn *β* = 0.28, winter *β* = 0.31, all *p* s < 0.001). A significant association with coastal-blue space visits was found in summer (*β* = 0.23), autumn (*β* = 0.21) and winter (*β* = 0.20; *p*s < 0.01), and with inland-blue space visits only in spring *β* = 0.14 and winter *β* = 0.14 (*ps* < 0.05). Nature connectedness was also only positively associated with WHO-5 in summer (*β* = 2.41), autumn (*β* = 2.29), and winter (*β* = 3.18; all *p*s < 0.05).

### Country-specific results (RQ2)

The stratified results predicting WHO-5 positive well-being for each country are presented in Supplementary Table S5. Results reflect the country-level heterogeneity identified through the random effect term in the main model. In terms of residential exposure, WHO-5 scores were significantly higher in both Ireland (Q2 *vs*. Q1: *β* = 4.20; Q3 *vs*. Q1: *β* = 4.15; Q4 *vs*. Q1: *β* = 3.65, *p*s < 0.05) and Italy (Q3 *vs*. Q1: *β* = 4.82; Q4 *vs*. Q1:* β* = 4.54, *p*s < 0.05) in greener neighbourhoods. This pattern was reversed for Finland (Q3 vs. Q1: *β* =  − 4.20, *p* < 0.05), where instead, having inland water within 1000 m was associated with significantly higher WHO-5 scores (*β* = 3.53, *p* < 0.01). By contrast, in Portugal, inland water was associated with significantly lower scores (*β* = -3.81, *p* < 0.05). Ireland was the only country where living within 1000 m of the coast was associated with higher WHO-5 scores when controlling for visits and connectedness (*β* = 5.00, *p* < 0.05).

An increase of one green space visit in the last four weeks was associated with significantly greater (at least *p* < 0.05) WHO-5 scores in Australia (*β* = 0.41), Bulgaria (*β* = 0.48), California (*β* = 0.42), Czech Republic (*β* = 0.27), Estonia (*β* = 0.23), Finland (*β* = 0.19), Greece (*β* = 0.54), Ireland (*β* = 0.39), Netherlands (*β* = 0.18), Portugal (*β* = 0.32), and Sweden (*β* = 0.32). For each extra inland-blue visit, WHO-5 scores were significantly higher (at least *p* < 0.05) in Germany (*β* = 0.36), Hong Kong (*β* = 0.53) and Spain (*β* = 0.44), and each additional coastal visit was associated with higher WHO-5 scores in France (*β* = 0.57), Portugal (*β* = 0.27), Spain (*β* = 0.24), and Sweden (*β* = 0.46). Finally a one-point increase in INS scores was associated with significantly higher (at least *p* < 0.05) WHO-5 scores in Canada (*β* = 4.30), Czech Republic (*β* = 5.41), Greece (*β* = 4.40), Hong Kong (*β* = 7.61), and UK (*β* = 3.59).

**Figure 3:**
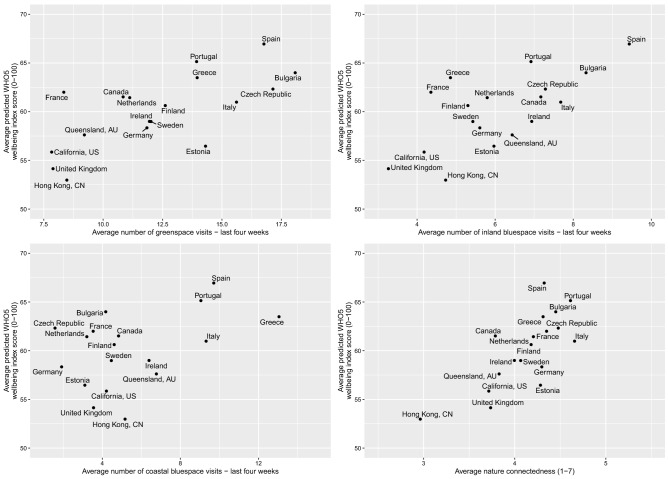
Country level relationships between positive well-being (0–100) and: (**a**) Green space visits in last 4 weeks; (**b**) Inland-blue space visits in last 4 weeks; (**c**) Coastal-blue space visits in last 4 weeks and (**d**) nature connectedness (1–7). Plots are based on aggregated predicted values across countries from our original mixed models controlling for residential exposure visit frequencies to alternative locations, connectedness (**a**–**c**), age, gender, employment status, household income, longstanding-illness, relationship status, education level, household composition, dog ownership, car ownership, physical activity, and season (sample wave).

To help visualise cross-country patterns we used the observed values from recreational visit frequency and connectedness, and the predicted values of WHO-5 from our original models, averaged across all individuals in each country (Fig. 3). With lower than average visit duration and connectedness, Hong Kong, the UK, and California, also reported the lowest positive well-being. By contrast, countries with the highest levels of positive well-being (e.g. Spain, Portugal, and Bulgaria) were among the countries with the highest nature visits and connectedness.

## Discussion

The present research provides significant new insights into the relationships between mental health, residential and recreational exposure to green and blue spaces, and feeling psychologically connected to the natural world. Collecting data in four seasonal waves, across 18 different countries/regions allowed us to make far more nuanced conclusions than are generally possible.

Contrary to Hypothesis 1, there was little evidence in the current sample that the amount of green, and presence of inland- and coastal-blue space, within 1000 m of the home was directly related to mental health. In models without recreational visits, but controlling for socio-demographic confounders, residents of the greenest and coastal areas did report higher positive well-being, but these effects disappeared when visits were added, suggesting that visit frequency mediated these effects. In other words, the reason why residents of the greenest and coastal neighbourhoods experienced better positive mental health might be because these neighbourhood qualities encouraged more frequent recreational visits^12, 14, 15^. The only residential exposure metric that significantly predicted positive mental health controlling for visits was living in the 3rd versus 1st quartile of green space, with the season models suggesting this was only significant in spring.

Despite the overall picture, some residential associations did remain after controlling for visits in the country-specific models. Ireland showed higher WHO-5 scores for those in greener and coastal neighbourhoods, and Italians also had higher positive well-being in greener neighbourhoods even accounting for visits and connectedness. Residents in Finland were the only sample to show significantly lower well-being in the greenest areas, though they did have higher well-being if they lived near rivers/lakes. Finally, those in Portugal had lower WHO-5 if they lived near inland waters. Although tempting, we are reluctant to speculate here about possible reasons for these cross-country differences. Our effect sizes are small, and thus some countries may not be showing patterns due to a lack of power. In countries where effects did emerge, we were not able to explore potential mechanisms underlying relationships. Further cross-country research is needed with larger within-country samples and a greater focus on potential mechanisms to address these possibilities, but the cross-country heterogeneity does support the contention that caution is needed when trying to generalise across locations^12^.

Supporting Hypothesis 2, the frequency of visits to green spaces in the last 4 weeks was positively associated with positive well-being and negatively associated with mental distress and the use of doctor-prescribed depression (though not anxiety) medication. Extending previous research, those who made more frequent visits to both inland- and coastal- blue spaces also reported more positive well-being and lower rates of mental distress, even controlling for the number of green space visits in the past four weeks. We recognise that despite being significant, these effects are, however, small in absolute terms. For instance, an extra 4 green space visits (i.e. one per week) is still only associated with a 1.04% higher WHO-5 score (i.e. *β* = 0.26 × 4, on the 100-point WHO-5 scale). Intriguingly, visiting inland-blue spaces was positively associated with anxiety medication use. Given that we asked about voluntary recreational visits, it seems unlikely that visits could *lead* to greater anxiety sufficient to require medication (or these individuals would stop going). Rather, we suspect that it reflects people with anxiety seeking these places out for the calming effects they have, and thus using them for self-management purposes^45, 46^.

Although visits decreased in frequency in autumn/winter, compared to spring/summer, the drop was not substantial and was similar for both green and blue spaces. Indeed, positive well-being remained significantly positively associated with visiting inland and coastal waters in winter, suggesting that potential benefits to mental health do not only occur in the warmer months. In terms of country-level effects, a positive association was found between at least one type of visit and WHO-5 scores in 16/18 countries, with no associations present for Canada or the UK. Most countries (11/18) showed a positive association with green space visit frequency, and Spain, Hong Kong and Germany showed a positive relationship with visits to inland waters. Much of the research on inland-blue spaces has come from Germany-based researchers^20, 23, 47^ potentially pointing to something more fundamental in a country with a relatively low coastline to population ratio. Three of the four countries showing a positive association between coastal visit frequency and mental health were in the warmer European South (Spain, Portugal and France). The fourth country to show this relationship was Sweden, which also showed a significant positive association with green space visits, potentially indicating the importance of overall outdoor nature recreation among this population for mental health^37^.

Partially supporting Hypothesis 3 there was also tentative evidence of non-linear relationships for visits, with diminishing marginal returns. Nevertheless, due the cross-sectional nature of the data, and small effect sizes, we remain cautious. Further work is needed including longitudinal work that follows people’s exposure over time and experimental work that randomly allocates people to different visit frequencies within a given period.

Supporting Hypothesis 4, greater nature connectedness was positively associated with positive well-being and negatively associated with both mental distress and depression medication use. When stratified by season and country (for WHO-5) a more complicated picture emerged. Although the association between connectedness and positive well-being was evident in summer, autumn and winter, it was non-significant in spring. By contrast, we note that residential greenness was *only* related to WHO-5 in spring and it may be that these are interacting in some way but we were unable to explore this further here. Combined, the findings suggest that researchers with quite a broad spectrum of interests in the nature-health field (inc. residential exposure, visits, connectedness) might want to be more sensitive to issues of seasonality in future work.

Further, only four countries showed significant associations between positive well-being and nature connectedness in the stratified models, two of which, Canada and the UK, were the only countries to not show significant associations between positive well-being and at least one sort of visit. While recognising the potential for statistical artefacts (due to shared variance), as with Germany and inland-blue space research, we note a strong research tradition in nature connectedness in both Canada^34^ and the UK^48^. Again we wonder whether this is merely coincidence or whether it reflects a higher level of importance of nature connectedness in these countries that has filtered through to research priorities, perhaps because of the relatively low levels of connectedness at the population level.

Intriguingly, the other countries/regions with low levels of connectedness (and visit frequencies), Hong Kong, California, Queensland, and Ireland, have certain commonalities in terms of language and cultural heritage with UK/Canada. Although there are very few international studies with which to compare our findings, Kruize et al.^49^ also found the lowest amount of regular time in nature in the UK city (Stoke on Trent) of their four city study (Barcelona [Spain], Kaunas [Lithuania], and Doetinchem [Netherlands]), supporting the current visit results. Further research is needed to explore what other commonalities these countries might have (e.g. economic models of growth or attitudes towards the natural environment) that could explain these findings.

Despite the robust sample and use of multiple, internationally recognised measures of well-being and mental health, we recognise several limitations with the current work. First, we acknowledge that multiple residential buffers have been used in past research, and it may be that the relatively little evidence of an association between residential exposure and mental health here is in part a consequence of our 1000 m selection based on a 10–15 min walk^50^. Further there may be limitations in the methods we used to establish common green/blue space residential metrics across European and non-European countries, or the way in which we operationalised green and blue spaces with these metrics (e.g. the landcovers we included in green space)^51^. Future international studies may want to select alternative buffers and/or methods of assessing residential exposure.

Second, much of the data were self-reported and we were unable to validate, for instance, people’s nature experiences or medication use. For current purposes, we applied approximate numerical values to verbal visit frequency response categories and it is also possible that some respondents ‘double-counted’ some visit locations (e.g. saying they had visited woodlands and a lake in the last 4 weeks when in fact they only did one visit that included both features). Similarly, although our prescription item is widely used^42^, it also does not account for length of use or dosage. Although challenging to collect on a similar scale as our multi-country study, more objective data on time in nature, e.g. using experience sampling approaches^22^, and mental health status should be a goal of future research.

Third, as already noted, the data is cross-sectional and thus can only speak to associations rather than causation. This was perhaps most evident in the positive association between inland-blue space visits and anxiety medication, which we took to suggest reverse causality. Nonetheless, many of our results are consistent with a growing body of experimental and longitudinal research, and used the sort of sample that would not be easily possible with these approaches.

Fourth, our results focus on averages and we recognise that individuals may vary widely in terms of the amount of nature that may benefit them personally, and that this too is likely to change over time as a function of need^45^.

Fifth, although our sample was collected by an international polling company and was weighted to be representative by age, gender and region within each country, it was not fully representative of the respective countries, in part due to limitations of online panels^52^. Our country-level observations therefore remain tentative at this stage.

Finally, our sample was limited to a selection of high-income countries/regions, and further research is needed in low-middle income nations where contact with the natural world, and consequent relationships, may be different. At this stage, our findings only speak to relatively developed settings where, typically, the natural world presents few threats and challenges. Conclusions about whether contact with, and connectedness to, the natural world is a universal good for human mental health and well-being will depend on the results of similar research across a far broader range of contexts.

These limitations notwithstanding, our findings have a number of implications. Results suggest the associations between recreational nature contact and clinical levels of mental distress are complicated. People may be using these environments to manage symptoms^46^ and perhaps we should not necessarily expect higher levels of recreational contact to be associated with incidence of depression and/or anxiety at a population level. More research is needed into how people with poor mental health spontaneously use nature to help with self-management, alongside more traditional research trying to support them to access these places through things such as ‘green prescriptions’^53^.

Results also offer support for initiatives e.g. education programs, aimed at increasing levels of psychological connectedness to the natural world, irrespective of direct exposure, for mental health as well as ecological reasons^54^. Given how relatively disconnected from the natural world our UK sample was, alongside low levels of well-being, it is promising that the UK government is prioritising the building of nature connectedness in the population^55^. Other countries in the English speaking world with low nature connectedness and well-being might consider a similar approach.

Finally, the results suggest that spending recreational time in both green and blue settings may be more important than merely living near nature, at least in terms of mental health. Although social inequalities in access and quality remain^56^, over 90% of people living in urban areas of Europe already have access to a public green space > 0.25 hectares within a 10-min walk of their home^57^. Promoting greater use of these green (and blue) spaces may be a policy objective to go alongside structural changes in the amount of green and blue spaces in people’s neighbourhoods. For instance, the United Nations (UN) Sustainable Development Goal [SDG] 11.7 proposes that “by 2030, [states should] provide universal access to safe, inclusive and accessible, green and public spaces, particularly for women and children, older persons and persons with disabilities”^58^. Future SDGs, or similar programs, might consider expressing targets in terms of use of, as well as access to, green/blue spaces, analogous to how SDG 12: ‘Ensure sustainable consumption and production patterns’, has sub-goals for both policies and infrastructure (12.1), and citizen actions and behaviors (12.5).

## Materials and methods

### Sample and survey

Data came from an 18-country self-report survey conducted as part of the BlueHealth project^59^, exploring recreational use of the natural environment with a particular focus on aquatic, or blue space, environments such as rivers, lakes and seas. It was administered by an international polling company using established online panels in four seasonal waves between June 2017 and April 2018. Stratified samples of ≈ 1000 respondents were collected in 14 European countries (Bulgaria, Czech Republic, Estonia, Finland, France, Germany, Greece, Ireland, Italy, Netherlands, Portugal, Spain, Sweden, and the United Kingdom) and four other countries/regions (California [USA], Canada, Hong Kong [China], and Queensland [Australia]). Stratified sampling by sex, age, and region of residence was undertaken to achieve broad national representativeness. The full sample consisted of 18,838 respondents, and survey weights were provided by data collectors to adjust for representativeness in analyses. Due to missing data (e.g. ‘don’t know’ responses on the INS scale and elsewhere) the analytical sample was *n* = 16,307. Full methodological details are available on the Open Science Framework website: https://doi.org/10.17605/OSF.IO/7AZU2^51^. Data collection was carried out in accordance with relevant guidelines and regulations, and informed consent was obtained from all participants. Ethical approval was granted by the University of Exeter Medical School’s Research Ethics Committee (Ref: Aug16/B/099).

### Mental health

Following previous research in the field^38, 39^, our measure of *positive well-being* was the World Health Organisation 5-item wellbeing index (WHO-5). Participants responded to five statements about their emotional state during the past two weeks e.g. “I have felt calm and relaxed”, on scales from ‘At no time’ (0) to ‘All of the time’ (5). Values were summed and multiplied by 4 to give a score out of 100, with higher scores reflecting higher well-being. An advantage of the WHO-5 is that scores < 28 have shown concurrent validity with structured clinical interviews for diagnosing depression/anxiety^40, 41^, and thus this threshold provided our first indicator of poor mental health, i.e. *mental distress*.

Our second and third indicators of poor mental health were self-reported use of doctor-prescribed medication for: (a) depression, and (b) anxiety. Respondents were asked: “During the past two weeks, have you used any medicines for any of the following conditions that were prescribed for you by a doctor? Please select all that apply”, with ‘yes’/‘no’ response options. Alongside physical health conditions, e.g. high blood pressure, were the conditions of current interest: ‘depression’ and ‘tension and anxiety’. The question was taken from the European Health Interview Survey^42^. As 4.0% (*n* = 740) reported taking both medications, our regressions predicting either outcome, controlled for concurrent use of the alternative medication type to identify the unique associations with contact and connectedness with use of each medication.

### Residential exposure

Participants were asked to input their home location via a Google Maps application programming interface. For confidentiality reasons, recorded coordinates were rounded to three decimal degrees on both the longitude and latitude scale. Residential natural environment exposure indicators were assigned to these coordinates using the Global Land Cover dataset (GlobeLand30), which is a globally-consistent 30 m resolution raster data set based on classification of remotely-sensed data. Full details of our processing of this data and references to relevant earlier work can be found in the technical report^51^. The data feature ten land cover classes which have demonstrated satisfactory congruence with more localised land use maps (general accuracy level of > 80%). Land classified as “forests”, “grassland”, “shrubland” and “cultivated land” was collapsed into a ‘green space’ measure and land classified as “water bodies” or “wetlands” into an ‘inland-blue space’ measure. Radial buffers of 1000 m around residential locations, representing a 10–15 min walk^50^ were established and the percentage of green and inland-blue spaces within these buffers assigned. Residential green space was divided into four quartiles, and due to a highly skewed distribution^15^, inland-blue space was categorised into just “none” = 0% (reference) and “some” > 0% to 100%. Residential exposure to coastal-blue space within 1000 m was calculated using a Euclidean (crow-flies) distance metric. Distance from the home coordinate to the nearest coastline was defined by the highest resolution version of the Global Self-consistent Hierarchical High-resolution Geography shoreline database from the National Oceanic and Atmospheric Administration^51^. This dataset provides a balance between refinement in capturing a good representation of the land-sea interface, but enough granularity that smaller rivers and other inland waterways are rarely miss-classified as coastline.

### Recreational contact with green/blue spaces

Participants were presented with a list, and archetypical pictures of, 12 types of green spaces (e.g. local park, woodlands, meadows), 9 inland-blue spaces (e.g. lake, rural river, canal) and 8 coastal-blue spaces (e.g. esplanades, rocky shores, beaches) and asked how often in the last 4 weeks they had visited each type of location. The last 4 weeks was chosen as an appropriate recall period due to its use in previous leisure visit surveys^51^. Response options, were: “Not at all in the last 4 weeks”, “Once or twice in the last 4 weeks”, ” Once a week” and “Several times a week”. For current purposes we estimated a numerical equivalent of these response options to be zero, one, four and eight visits in the last 4 weeks respectively.

Total green space visits in the last 4 weeks were derived by summing the visit frequency estimates for each of the 12 green space types. Due to a small number of people reporting very high visit frequencies, and introducing considerable skew, we capped the total number of visits to 56, which would be consistent with someone, for instance, walking their dog twice a day over a 4-week period. Only 1.5% of respondents were capped in this way. Four weekly inland- and coastal-blue space visit frequencies were derived in a similar way with only 0.5% and 0.6% of respondents requiring a cap for inland and coastal visits respectively.

### Nature connectedness

Psychological connectedness to the natural world was measured using the Inclusion of Nature in Self (INS) scale^43, 44^. Seven images were presented with two circles, one labelled ‘Self’ and one labelled ‘Nature’, which increasingly overlapped with each image to indicate greater nature connectedness. Participants were asked to select the picture “that best describes your relationship with the natural environment. How interconnected are you with nature?” with the lowest connectedness reflecting no overlap between the circles (1), and highest connectedness reflecting almost totally overlapping circles (7).

### Covariates

Sociodemographic controls, comparable to related studies, included: gender (female = *ref*; male); age (16–29 years = *ref*; 30–39 years; 40–49 years; 50–59 years; ≥ 60 years); highest educational achievement (degree; below degree = *ref*); employment status (in paid employment, in education, retired, homemaker; not working/unemployed = *ref*); disposable household income quintiles (lowest quintile = *ref*); longstanding illness or disability (i.e. underlying health condition, yes, no = *ref*); relationship status (married/cohabiting; single/separated/divorced/widowed = *ref*); number of adults in the household (1 = *ref;* 2, ≥ 3); number of children in the household (0 = *ref;* 1, ≥ 2); dog ownership (yes, no = *ref*); car ownership (yes, no = *ref*); weekly days of physical activity ≥ 30 min (0 = *ref*, 1–4, ≥ 5); and survey wave (spring = *ref*, summer, autumn, winter). Of note seasons were approximate since ‘Spring’ data were collected in June and referred to the ‘last 4 weeks’ (i.e. May–June), ‘Summer’ in September (i.e. August–September), ‘Autumn’ in December (November to December), and ‘Winter’ in March (i.e. February–March), seasons were reversed for Australia. Again, full details are available in the technical report online^51^.

### Analyses

Hypotheses were tested using a series of linear mixed effects models for WHO-5 scores, and Bernoulli generalised linear mixed effects models for the binary outcome variables of mental distress and use of medication for depression and anxiety. Models included quadratic (squared) terms for visit frequency and connectedness to test for non-linearity (diminishing marginal returns)^19^. Country of residence was included as a random intercept term to account for national-level respondent clustering. Models were fitted by maximum likelihood with Laplace approximation (to integrate the random effects), and survey weights were applied to improve national representativeness with regards to the sampling strata within each country (sex, age, and region of residence). Analyses controlled for covariates listed above, with models for depression medication also controlling for anxiety medication and vice versa. Each dependent variable was analysed using three models: (a) residential exposure and covariates only, (b) residential, covariates plus recreational contact; and (c) residential, covariates, recreational plus connectedness. This allowed us to see how the addition of recreation and connectedness affected residential relationships. The largest generalized variance inflation factor (VIF) of any term in any of the fully-adjusted models was VIF = 1.81, suggesting there was no substantive multi-collinearity in any of the models. All models are presented in Supplementary Tables S2 and S3 and only the final models including all exposure measures are in the main text due to space constraints (Table 2). The full WHO-5 model was subsequently stratified by season and country to explore potential variation across the year and location. We did not perform similar stratifications for mental distress or medication use due to lack of power in predicting these binary outcomes in stratified models. Analyses were performed in R v3.6.0 (R Core Team, 2019) using the ‘lme4’ package for statistical modelling^60^.

## Supplementary Information

Supplementary Information.

## Data Availability

All data for the BlueHealth International Survey will be made open access in 2025 in accordance with an embargo agreement by research partners. For queries about the specific data and analysis, including r script, used in the present manuscript please contact the corresponding author.
