# Supplementary material for: Associations between green/blue spaces and mental health across 18 countries
Source: Sci Rep. 2021 Apr 26;11:8903. doi: 10.1038/s41598-021-87675-0 (PMC8076244; doi:10.1038/s41598-021-87675-0)

**Supplementary Materials**

# **Associations between green/blue spaces and mental health across 18 countries**

Mathew P. White^1,2^*, Lewis R. Elliott^2^, James Grellier^2,3^, Theo Economou^4^, Simon Bell^5^, Gregory N. Bratman^6^, Marta Cirach^7,8,9^, Mireia Gascon^7,8,9^, Maria L. Lima^10^, Mare Lõhmus^11^, Mark Nieuwenhuijsen^7,8,9^, Ann Ojala^12^, Anne Roiko^13^, P. Wesley Schultz^14^, Matilda van den Bosch^7,15,16^ & Lora E. Fleming^2^

*Corresponding author: mathew.white@univie.ac.at; Liebbigasse 5, Cognitive Science HUB, University of Vienna, Vienna, 1110 Austria

^1^ Cognitive Science HUB, University of Vienna, Vienna, Austria

^2^ European Centre for Environment and Human Health, University of Exeter Medical School, United Kingdom

^3^ Institute of Psychology, Jagiellonian University, Krakow, Poland

^4^ College of Engineering, Mathematics, and Physical Sciences, University of Exeter, United Kingdom

^5^ Estonian University of life Sciences, Tartu, Estonia

^6^ School of Environmental and Forest Sciences, College of the Environment, University of Washington, USA

^7^ ISGlobal, Barcelona, Spain

^8^ Universitat Pompeu Fabra (UPF), Barcelona, Spain

^9^ CIBER Epidemiología y Salud Pública (CIBERESP), Madrid, Spain

^10^ Department of Social and Organizational Psychology, ISCTE – University Institute of Lisbon, Portugal

^11^ Institute of Environmental Medicine, Karolinska Institute, Sweden

^12^ Natural Resources Institute Finland (Luke), Finland

^13^ School of Medicine, Griffith University, Australia

^14^ Department of Psychology, California State University San Marcos, USA

^15^ School of Population and Public Health, University of British Columbia, Canada

^16^ Department of Forest and Conservation Sciences, University of British Columbia, Canada

**Descriptives**

Mean *positive well-being* as measured by the WHO-5 was slightly lower *M* = 60.16 (*SD* = 21.72) than previous European findings (*M* = 64.44) albeit with a different range of countries^1^_,_ and 8.70% (*n* = 1,418) were classified as being in *mental distress* using the <28 threshold. Approximately 8.90% (n = 1,451) of participants reported taking medication for depression and 9.48% (*n* = 1,545) for tension/anxiety, with 4.09% (*n* = 667) for both conditions. The combined total of 14.29% (*n* = 2,329) for either drug is a little less than a 17% global estimate of common mental health disorders^2^. The average amount of green space within a 1000m radius of home rose from *M* = 1.36% to 19.79%, 62.11% and 96.85% across the four quartiles respectively (Table 1). Thirty-seven percent of people had some inland-blue space within 1000m of their home but only 11% were within 1000m of the coast. The mean number of green space visits in the last 4 weeks was 12.34 (i.e. just over 3/week on average), and the number of blue space visits was slightly lower M = 11.42 (6.08 inland; 5.34 coastal). Although the correlations between visit frequency across the three locations were all significantly positive (green-inland *r* = 0.67, *p* < 0.001; green-coastal *r* = 0.42, *p* < 0.001; inland-coastal *r* = 0.49, *p* < 0.001) none were so high (e.g. > 0.80) that we might expect multi-collinearity. Mean psychological *connectedness,* using the 7-point INS scale, was *M* = 4.14.

Bivariate correlations (Table 1), showed that visit frequencies to all three settings and nature connectedness were independently positively correlated with positive well-being (WHO-5: *r*s = 0.18 to 0.26; *p*s < 0.001) and negatively correlated with mental distress (WHO-5 < 28: point-biserial *r_pb_*s = -0.07 to -0.12; *p*s < 0.001). However, visit frequency to all three spaces was positively associated with anxiety medication use (*r_pb_*s = 0.03 to 0.06; *p*s < 0.001). Nature connectedness was negatively associated with the likelihood of using depression (*r_pb_* = -0.04, *p* < 0.001) and anxiety (*r_pb_* = -0.03, *p* < 0.001) medication.

**Covariates**

Supporting the robustness/appropriateness of the models, results for covariates replicated previous findings for gender, age, income, employment status, education, long-term illness, marital status, car ownership, and physical activity^3^ (see Supplementary Tables S2 & S3). Of note, there were no effects of season at this point. In terms of country ranks, medication use was broadly similar to Eurobarometer data^4^ (Supplementary Figure 1), however the WHO-5 order was somewhat different, with Greece and Portugal on average reporting the highest WHO-5 scores, which have appeared lower in earlier findings^5^. Fully adjusted models including country as a random effect explained between 23-25% of the variance in outcomes.

**References**

1. Bericat, E. (2019). Subjective Well-Being. In E. Bericat & M.L. Jimenez Rodrigo (Eds) *The Quality of European Societies* (pp. 75-91). Springer, Cham, Switzerland.
2. Steel, Z. *et al.* (2014). The global prevalence of common mental disorders: a systematic review and meta-analysis 1980-2013. *Int. J. Epidemiol.* **43,** 476–493.
3. Dolan, P., Peasgood, T., & White, M. (2008). Do we really know what makes us happy? A review of the economic literature on the factors associated with subjective well-being. *J. Econ. Psychol.*, ***29*,** 94-122.
4. Bericat, E. (2019). Subjective Well-Being. In E. Bericat & M.L. Jimenez Rodrigo (Eds) *The Quality of European Societies* (pp. 75-91). Springer, Cham, Switzerland.
5. Lewer, D., O'Reilly, C., Mojtabai, R., & Evans-Lacko, S. (2015). Antidepressant use in 27 European countries: associations with sociodemographic, cultural and economic factors. *Brit. J. Psychiat.*, **207,** 221-226.

**Supplementary Table S1**: The Ns, Percentages (%), Means (Ms), Standard Deviations (SDs), and correlations (r = Pearson’s; r_pb_ = Point-biserial) and for the four mental health outcomes as a function of residential exposure, recreational visits, nature connectedness, and covariates for the analytical sample (n = 16,302).

|  |  |  |  |  | WHO-5 | | WHO-5 < 28 | | Depression Meds | | Anxiety Meds | |
| --- | --- | --- | --- | --- | --- | --- | --- | --- | --- | --- | --- | --- |
|  | n | % | M | SD | M / r | SD | N / r_pb_ | % | N / r_pb_ | % | N / r_pb_ | % |
| ***Residential exposure [within 1000m]*** |  |  |  |  |  |  |  |  |  |  |  |  |
| Greenspace [Q1] | 4103 | 25.17 | 1.36 | 1.87 | 58.79 | 21.55 | 381 | 9.29 | 354.00 | 8.63 | 366.00 | 8.92 |
| Greenspace [Q2] | 4098 | 25.14 | 19.79 | 9.35 | 59.73 | 21.57 | 352 | 8.59 | 362.00 | 8.83 | 389.00 | 9.49 |
| Greenspace [Q3] | 4071 | 24.97 | 62.11 | 14.44 | 61.29 | 21.53 | 333 | 8.18 | 330.00 | 8.11 | 374.00 | 9.19 |
| Greenspace [Q4] | 4030 | 24.72 | 96.85 | 4.18 | 60.86 | 22.17 | 352 | 8.73 | 405.00 | 10.05 | 416.00 | 10.32 |
| Inland blue [No] | 10141 | 62.21 | NA | NA | 60.27 | 22.00 | 897 | 8.85 | 872.00 | 8.60 | 962.00 | 9.49 |
| Inland blue [Yes] | 6161 | 37.79 | NA | NA | 59.98 | 21.25 | 521 | 8.46 | 579.00 | 9.40 | 583.00 | 9.46 |
| Coastal blue [No] | 14507 | 88.99 | NA | NA | 60.04 | 21.77 | 1272 | 8.77 | 1330.00 | 9.17 | 1410.00 | 9.72 |
| Coastal blue [Yes] | 1795 | 11.01 | NA | NA | 61.11 | 21.35 | 146 | 8.13 | 121.00 | 6.74 | 135.00 | 7.52 |
| ***Visits***  ***[last 4 weeks]*** |  |  |  |  |  |  |  |  |  |  |  |  |
| Green | NA | NA | 12.34 | 12.85 | 0.26^***^ | NA | -0.12^***^ | NA | -0.01 | NA | 0.03^***^ | NA |
| Inland blue | NA | NA | 6.08 | 8.95 | 0.19^***^ | NA | -0.08^***^ | NA | 0.03^***^ | NA | 0.06^***^ | NA |
| Coastal blue | NA | NA | 5.34 | 10.17 | 0.18^***^ | NA | -0.07^***^ | NA | 0.00 | NA | 0.05^***^ | NA |
| ***Nature Connectedness*** |  |  |  |  |  |  |  |  |  |  |  |  |
| INS | NA | NA | 4.14 | 1.65 | 0.24^***^ | NA | -0.11^***^ | NA | -0.04^***^ | NA | -0.03*^***^ | NA |
| ***Socio-demographics*** |  |  |  |  |  |  |  |  |  |  |  |  |
| Female | 8298 | 50.90 | NA | NA | 59.06 | 22.05 | 790 | 9.52 | 813.00 | 9.80 | 871.00 | 10.50 |
| Male | 8004 | 49.10 | NA | NA | 61.31 | 21.32 | 628 | 7.85 | 638.00 | 7.97 | 674.00 | 8.42 |
| Age yrs [18-29] | 3085 | 18.92 | NA | NA | 56.87 | 21.55 | 302 | 9.79 | 275.00 | 8.91 | 330.00 | 10.70 |
| Age yrs [30-39] | 2997 | 18.38 | NA | NA | 59.01 | 22.10 | 284 | 9.48 | 254.00 | 8.48 | 302.00 | 10.08 |
| Age yrs [40-49] | 3044 | 18.67 | NA | NA | 59.90 | 21.87 | 276 | 9.07 | 299.00 | 9.82 | 312.00 | 10.25 |
| Age yrs [50-59] | 2956 | 18.13 | NA | NA | 59.72 | 21.87 | 276 | 9.34 | 327.00 | 11.06 | 321.00 | 10.86 |
| Age yrs [60+] | 4220 | 25.89 | NA | NA | 63.89 | 20.84 | 280 | 6.64 | 296.00 | 7.01 | 280.00 | 6.64 |
| HH income [Q1 = Low] | 2562 | 15.72 | NA | NA | 54.12 | 23.15 | 382 | 14.91 | 388.00 | 15.14 | 361.00 | 14.09 |
| HH income [Q2] | 2410 | 14.78 | NA | NA | 58.45 | 22.69 | 251 | 10.41 | 273.00 | 11.33 | 270.00 | 11.20 |
| HH income [Q3] | 2563 | 15.72 | NA | NA | 61.08 | 21.02 | 198 | 7.73 | 211.00 | 8.23 | 244.00 | 9.52 |
| HH income [Q4] | 2836 | 17.40 | NA | NA | 62.36 | 20.64 | 182 | 6.42 | 215.00 | 7.58 | 248.00 | 8.74 |
| HH income [Q5 = High] | 3706 | 22.73 | NA | NA | 63.52 | 20.36 | 206 | 5.56 | 231.00 | 6.23 | 273.00 | 7.37 |
| HH income  [prefer not answer] | 2225 | 13.65 | NA | NA | 59.52 | 21.67 | 199 | 8.94 | 133.00 | 5.98 | 149.00 | 6.70 |
| Unemployed | 2283 | 14.00 | NA | NA | 52.46 | 24.12 | 383 | 16.78 | 397.00 | 17.39 | 360.00 | 15.77 |
| Retired | 3050 | 18.71 | NA | NA | 64.22 | 20.75 | 211 | 6.92 | 250.00 | 8.20 | 240.00 | 7.87 |
| Home maker | 886 | 5.43 | NA | NA | 58.26 | 22.54 | 103 | 11.63 | 72.00 | 8.13 | 91.00 | 10.27 |
| In education | 1076 | 6.60 | NA | NA | 57.63 | 20.96 | 83 | 7.71 | 84.00 | 7.81 | 98.00 | 9.11 |
| Employed | 9007 | 55.25 | NA | NA | 61.23 | 20.88 | 638 | 7.08 | 648.00 | 7.19 | 756.00 | 8.39 |
| University ed. [No] | 7925 | 48.61 | NA | NA | 58.76 | 22.22 | 798 | 10.07 | 801.00 | 10.11 | 820.00 | 10.35 |
| University ed.[Yes] | 8377 | 51.39 | NA | NA | 61.48 | 21.16 | 620 | 7.40 | 650.00 | 7.76 | 725.00 | 8.65 |
| Ill/disability [No] | 10343 | 63.45 | NA | NA | 64.12 | 19.97 | 549 | 5.31 | 292.00 | 2.82 | 449.00 | 4.34 |
| Ill/disability [Yes] | 5959 | 36.55 | NA | NA | 53.29 | 22.90 | 869 | 14.58 | 1159.00 | 19.45 | 1096.00 | 18.39 |
| Married [No] | 6633 | 40.69 | NA | NA | 56.79 | 22.56 | 774 | 11.67 | 749.00 | 11.29 | 728.00 | 10.98 |
| Married [Yes] | 9669 | 59.31 | NA | NA | 62.47 | 20.82 | 644 | 6.66 | 702.00 | 7.26 | 817.00 | 8.45 |
| Adults in HH [1] | 3754 | 23.03 | NA | NA | 57.46 | 22.72 | 442 | 11.77 | 478.00 | 12.73 | 433.00 | 11.53 |
| Adults in HH [2] | 8251 | 50.61 | NA | NA | 61.64 | 21.11 | 594 | 7.20 | 598.00 | 7.25 | 716.00 | 8.68 |
| Adults in HH [3+] | 4297 | 26.36 | NA | NA | 59.69 | 21.76 | 382 | 8.89 | 375.00 | 8.73 | 396.00 | 9.22 |
| Children in HH [0] | 11703 | 71.79 | NA | NA | 59.71 | 21.74 | 1072 | 9.16 | 1067.00 | 9.12 | 1068.00 | 9.13 |
| Children in HH [1] | 2491 | 15.28 | NA | NA | 61.33 | 21.66 | 187 | 7.51 | 211.00 | 8.47 | 262.00 | 10.52 |
| Children in HH[2+] | 2108 | 12.93 | NA | NA | 61.29 | 21.64 | 159 | 7.54 | 173.00 | 8.21 | 215.00 | 10.20 |
| Owns dog [no] | 11308 | 69.37 | NA | NA | 59.46 | 21.45 | 1004 | 8.88 | 911.00 | 8.06 | 955.00 | 8.45 |
| Owns dog [Yes] | 4994 | 30.63 | NA | NA | 61.74 | 22.25 | 414 | 8.29 | 540.00 | 10.81 | 590.00 | 11.81 |
| Owns car [no] | 2844 | 17.45 | NA | NA | 53.10 | 22.63 | 395 | 13.89 | 346.00 | 12.17 | 327.00 | 11.50 |
| Owns car [Yes] | 13458 | 82.55 | NA | NA | 61.65 | 21.23 | 1023 | 7.60 | 1105.00 | 8.21 | 1218.00 | 9.05 |
| Days active [0] | 4193 | 25.72 | NA | NA | 52.79 | 22.84 | 636 | 15.17 | 476.00 | 11.35 | 421.00 | 10.04 |
| Days active [1-4] | 8843 | 54.24 | NA | NA | 61.41 | 20.68 | 605 | 6.84 | 741.00 | 8.38 | 873.00 | 9.87 |
| Days active [5+] | 3266 | 20.03 | NA | NA | 66.26 | 20.43 | 177 | 5.42 | 234.00 | 7.16 | 251.00 | 7.69 |
| ***Season*** |  |  |  |  |  |  |  |  |  |  |  |  |
| Spring | 4122 | 25.29 | NA | NA | 60.89 | 21.49 | 333 | 8.08 | 368.00 | 8.93 | 384.00 | 9.32 |
| Summer | 3546 | 21.75 | NA | NA | 61.33 | 21.36 | 263 | 7.42 | 325.00 | 9.17 | 351.00 | 9.90 |
| Autumn | 4248 | 26.06 | NA | NA | 59.29 | 21.80 | 407 | 9.58 | 368.00 | 8.66 | 388.00 | 9.13 |
| Winter | 4386 | 26.90 | NA | NA | 59.37 | 22.10 | 415 | 9.46 | 390.00 | 8.89 | 422.00 | 9.62 |
| ***Medication use*** |  |  |  |  |  |  |  |  |  |  |  |  |
| Depression [No] | 14757 | 90.52 | NA | NA | 61.28 | 21.12 | 1105 | 7.49 | 784.00 | 5.31 | NA | NA |
| Depression [Yes] | 1545 | 9.48 | NA | NA | 49.49 | 24.36 | 313 | 20.26 | 667.00 | 43.17 | NA | NA |
| Anxiety [No] | 14851 | 91.10 | NA | NA | 61.57 | 21.00 | 1068 | 7.19 | NA | NA | 878.00 | 5.91 |
| Anxiety [Yes] | 1451 | 8.90 | NA | NA | 45.79 | 23.72 | 350 | 24.12 | NA | NA | 667.00 | 45.97 |

r = Pearson’s correlation; r_pb_ = Point bi-serial correlation (due to binary outcome); Q = quartile for greenspace and quintile for income; ^‡^ HH = Household; INS = Inclusion of Nature in Self Scale; Married includes cohabiting; Seasons are approximate since ‘Spring’ data were collected in June and referred to the ‘last 4 weeks’ (i.e. May-June), ‘Summer’ in September (i.e. August-September), ‘Autumn’ in December (November to December), and ‘Winter’ in March (i.e. February – March), seasons were reversed for Australia; * *p* < 0.001.

**Supplementary Table S2**: Models for WHO-5 Scale and WHO-5 < 28.

|  | **WHO-5 Scale (0-100)** | | | | | | **WHO-5 Distress (< 28)** | | | | | |
| --- | --- | --- | --- | --- | --- | --- | --- | --- | --- | --- | --- | --- |
|  | **Residential + covariates** | | **+ Visits** | | **+ INS** | | **Residential only + covariates** | | **+ Visits** | | **+ INS** | |
|  | *Estimates* | *95% CIs* | *Estimates* | *95% CIs* | *Estimates* | *95% CIs* | *Odds Ratios* | *95% CIs* | *Odds Ratios* | *95% CIs* | *Odds Ratios* | *95% CIs* |
| (Intercept) | 42.89 ^***^ | 40.48, 45.29 | 47.43 ^***^ | 45.25, 49.60 | 48.15 ^***^ | 46.06, 50.24 | 0.37 ^***^ | 0.26, 0.52 | 0.21 ^***^ | 0.15, 0.30 | 0.19 ^***^ | 0.14, 0.27 |
| ***Residential exposure [within 1000m]*** |  |  |  |  |  |  |  |  |  |  |  |  |
| Greenspace [Q2 vs. Q1] | 0.76 | -0.11, 1.63 | 0.51 | -0.33, 1.36 | 0.46 | -0.38  , 1.30 | 0.93 | 0.79, 1.09 | 0.93 | 0.79, 1.10 | 0.94 | 0.80, 1.11 |
| Greenspace [Q3 vs. Q1] | 1.99 ^***^ | 1.10, 2.87 | 1.14 ^**^ | 0.28, 2.01 | 1.01 ^*^ | 0.15, 1.87 | 0.87 | 0.73, 1.03 | 0.91 | 0.77, 1.08 | 0.93 | 0.78, 1.10 |
| Greenspace [Q4 vs. Q1] | 1.78 ^***^ | 0.87, 2.68 | 0.47 | -0.42, 1.35 | 0.37 | -0.51, 1.25 | 0.93 | 0.79, 1.10 | 1.02 | 0.86, 1.21 | 1.02 | 0.86, 1.21 |
| Inland blue [Yes vs. No] | 0.18 | -0.50, 0.86 | -0.00 | -0.67, 0.66 | -0.08 | -0.74, 0.58 | 0.94 | 0.82, 1.07 | 0.94 | 0.82, 1.07 | 0.94 | 0.82, 1.07 |
| Coastal blue [Yes vs. No] | 1.98 ^***^ | 0.93, 3.03 | 0.78 | -0.28, 1.84 | 0.74 | -0.31, 1.79 | 0.91 | 0.74, 1.12 | 1.01 | 0.82, 1.26 | 1.01 | 0.81, 1.25 |
| ***Visits***  ***[last 4 weeks]*** |  |  |  |  |  |  |  |  |  |  |  |  |
|  |  |  |  |  |  |  |  |  |  |  |  |  |
| Green | / | / | 0.32 ^***^ | 0.28, 0.37 | 0.26 ^***^ | 0.22, 0.30 | / | / | 0.97 ^***^ | 0.96, 0.98 | 0.97 ^***^ | 0.96, 0.98 |
| Green ^2^ | / | / | -0.00 ^***^ | -0.00, -0.00 | -0.00 ^*^ | -0.00, -0.00 | / | / | 1.00 ^**^ | 1.00, 1.00 | 1.00 ^*^ | 1.00, 1.00 |
| Inland blue | / | / | 0.14 ^***^ | 0.07, 0.21 | 0.12 ^***^ | 0.05, 0.19 | / | / | 0.97 ^***^ | 0.95, 0.99 | 0.97 ^***^ | 0.96, 0.99 |
| Inland blue ^2^ | / | / | -0.00 ^*^ | -0.01, -0.00 | -0.00 | -0.00, 0.00 | / | / | 1.00 ^**^ | 1.00, 1.00 | 1.00 ^**^ | 1.00, 1.00 |
| Coastal blue | / | / | 0.20 ^***^ | 0.13, 0.27 | 0.19 ^***^ | 0.12, 0.25 | / | / | 0.97 ^***^ | 0.95, 0.99 | 0.97 ^***^ | 0.96, 0.99 |
| Coastal blue ^2^ | / | / | -0.00 | -0.00, 0.00 | -0.00 | -0.00, 0.00 | / | / | 1.00 ^*^ | 1.00, 1.00 | 1.00 ^*^ | 1.00, 1.00 |
| ***Nature Connectedness*** |  |  |  |  |  |  |  |  |  |  |  |  |
| INS | / | / | / | / | 2.35 ^***^ | 1.45, 3.25 | / | / | / | / | 0.62 ^***^ | 0.52, 0.72 |
| INS^2^ | / | / | / | / | -0.09 | -0.20, 0.01 | / | / | / | / | 1.05 ^***^ | 1.03, 1.07 |
| ***Socio-demographics*** |  |  |  |  |  |  |  |  |  |  |  |  |
| Sex [Male vs. Female] | 0.81 ^*^ | 0.18, 1.43 | 0.17 | -0.44, 0.78 | 0.31 | -0.30, 0.92 | 0.93 | 0.83, 1.05 | 0.98 | 0.87, 1.11 | 0.97 | 0.86, 1.10 |
| Age [30-39 vs. 18-29] | -0.00 | -1.11, 1.10 | 0.26 | -0.81, 1.34 | 0.05 | -1.02, 1.12 | 1.13 | 0.93, 1.38 | 1.07 | 0.88, 1.31 | 1.09 | 0.89, 1.34 |
| Age [40-49 vs. 18-29] | 0.96 | -0.15, 2.07 | 1.82 ^***^ | 0.74, 2.91 | 1.45 ^**^ | 0.38, 2.53 | 1.01 | 0.83, 1.24 | 0.90 | 0.73, 1.10 | 0.92 | 0.75, 1.13 |
| Age [50-59 vs. 18-29] | 2.14 ^***^ | 1.02, 3.26 | 3.05 ^***^ | 1.96, 4.15 | 2.36 ^***^ | 1.27, 3.45 | 0.86 | 0.70, 1.06 | 0.77 ^*^ | 0.63, 0.95 | 0.82 | 0.66, 1.01 |
| Age [60+ vs. 18-29] | 5.52 ^***^ | 4.25, 6.79 | 6.00 ^***^ | 4.75, 7.24 | 5.18 ^***^ | 3.94, 6.42 | 0.57 ^***^ | 0.44, 0.73 | 0.53 ^***^ | 0.41, 0.68 | 0.56 ^***^ | 0.43, 0.72 |
| HH income [Q2 vs. Q1] | 1.73 ^**^ | 0.60, 2.87 | 1.58 ^**^ | 0.48, 2.69 | 1.50 ^**^ | 0.40, 2.60 | 0.80 ^*^ | 0.66, 0.96 | 0.81 ^*^ | 0.67, 0.98 | 0.82 ^*^ | 0.68, 1.00 |
| HH income [Q3 vs. Q1] | 2.47 ^***^ | 1.32, 3.63 | 2.45 ^***^ | 1.33, 3.58 | 2.58 ^***^ | 1.46, 3.69 | 0.70 ^***^ | 0.57, 0.86 | 0.70 ^***^ | 0.57, 0.87 | 0.71 ^**^ | 0.58, 0.87 |
| HH income [Q4 vs. Q1] | 3.16 ^***^ | 1.99, 4.33 | 3.06 ^***^ | 1.92, 4.20 | 3.13 ^***^ | 1.99, 4.26 | 0.63 ^***^ | 0.51, 0.79 | 0.64 ^***^ | 0.51, 0.80 | 0.65 ^***^ | 0.52, 0.81 |
| HH income [Q5 vs. Q1] | 4.90 ^***^ | 3.71, 6.10 | 4.76 ^***^ | 3.60, 5.93 | 4.92 ^***^ | 3.77, 6.08 | 0.54 ^***^ | 0.43, 0.68 | 0.55 ^***^ | 0.44, 0.69 | 0.54 ^***^ | 0.43, 0.68 |
| HH income [prefer not answer vs. Q1] | 2.45 ^***^ | 1.26, 3.63 | 2.61 ^***^ | 1.46, 3.77 | 2.68 ^***^ | 1.54, 3.83 | 0.74 ^**^ | 0.60, 0.91 | 0.73 ^**^ | 0.59, 0.89 | 0.74 ^**^ | 0.60, 0.91 |
| Retired [vs. Unemployed] | 6.03 ^***^ | 4.71, 7.35 | 5.75 ^***^ | 4.46, 7.04 | 5.75 ^***^ | 4.47, 7.02 | 0.65 ^***^ | 0.51, 0.82 | 0.65 ^***^ | 0.51, 0.83 | 0.65 ^***^ | 0.51, 0.83 |
| Home maker [vs. Unemployed] | 2.46 ^**^ | 0.86, 4.05 | 2.88 ^***^ | 1.32, 4.43 | 2.88 ^***^ | 1.33, 4.42 | 0.88 | 0.67, 1.14 | 0.84 | 0.65, 1.10 | 0.85 | 0.65, 1.12 |
| In education [vs. Unemployed] | 4.51 ^***^ | 2.95, 6.07 | 4.47 ^***^ | 2.95, 5.99 | 4.72 ^***^ | 3.21, 6.23 | 0.46 ^***^ | 0.35, 0.62 | 0.45 ^***^ | 0.34, 0.60 | 0.45 ^***^ | 0.33, 0.59 |
| Employed [vs. Unemployed] | 4.23 ^***^ | 3.25, 5.21 | 4.16 ^***^ | 3.20, 5.11 | 4.25 ^***^ | 3.30, 5.20 | 0.61 ^***^ | 0.52, 0.71 | 0.60 ^***^ | 0.51, 0.70 | 0.59 ^***^ | 0.50, 0.70 |
| University ed.[ Yes vs. No] | 0.35 | -0.30, 1.01 | 0.05 | -0.59, 0.69 | 0.04 | -0.60, 0.67 | 0.94 | 0.83, 1.07 | 0.97 | 0.85, 1.10 | 0.97 | 0.86, 1.11 |
| Ill/disability [Yes vs. No] | -9.24 ^***^ | -9.90, -8.57 | -10.04 ^***^ | -10.69, -9.39 | -9.90 ^***^ | -10.54, -9.25 | 2.65 ^***^ | 2.34, 3.00 | 2.77 ^***^ | 2.44, 3.14 | 2.74 ^***^ | 2.41, 3.10 |
| Married [Yes vs. No] | 2.06 ^***^ | 1.19, 2.93 | 1.43 ^***^ | 0.59, 2.28 | 1.16 ^**^ | 0.32, 2.00 | 0.77 ^**^ | 0.66, 0.91 | 0.80 ^**^ | 0.68, 0.94 | 0.82 ^*^ | 0.69, 0.96 |
| Adults in HH [2 vs. 1] | -0.49 | -1.48, 0.50 | -0.04 | -1.01, 0.93 | 0.04 | -0.92, 1.00 | 0.92 | 0.77, 1.09 | 0.91 | 0.76, 1.09 | 0.90 | 0.75, 1.07 |
| Adults in HH [3+ vs. 1] | -1.14 ^*^ | -2.17, -0.11 | -0.68 | -1.69, 0.32 | -0.61 | -1.61, 0.39 | 1.02 | 0.85, 1.22 | 1.01 | 0.84, 1.21 | 0.99 | 0.82, 1.19 |
| Children in HH [1 vs. 0] | 0.82 | -0.11, 1.75 | -0.28 | -1.19, 0.63 | -0.18 | -1.08, 0.72 | 0.96 | 0.80, 1.15 | 1.02 | 0.85, 1.23 | 1.00 | 0.83, 1.20 |
| Children in HH[2+ vs. 0] | 0.64 | -0.40, 1.67 | -0.62 | -1.63, 0.39 | -0.48 | -1.49, 0.52 | 0.95 | 0.77, 1.16 | 1.06 | 0.86, 1.30 | 1.03 | 0.84, 1.27 |
| Owns dog [Yes vs. No] | 1.48 ^***^ | 0.79, 2.18 | -0.21 | -0.90, 0.48 | -0.51 | -1.20, 0.17 | 1.01 | 0.88, 1.15 | 1.16 ^*^ | 1.01, 1.33 | 1.19 ^*^ | 1.04, 1.37 |
| Owns car [Yes vs. No] | 3.38 ^***^ | 2.48, 4.28 | 2.97 ^***^ | 2.09, 3.85 | 2.87 ^***^ | 2.00, 3.74 | 0.77 ^***^ | 0.66, 0.90 | 0.82 ^**^ | 0.71, 0.95 | 0.84 ^*^ | 0.73, 0.98 |
| Days active [1-4 vs. 0] | 7.60 ^***^ | 6.86, 8.35 | 5.28 ^***^ | 4.53, 6.02 | 4.84 ^***^ | 4.10, 5.58 | 0.46 ^***^ | 0.41, 0.53 | 0.58 ^***^ | 0.51, 0.66 | 0.61 ^***^ | 0.54, 0.70 |
| Days active [5+ vs. 0] | 11.38 ^***^ | 10.45, 12.32 | 7.70 ^***^ | 6.75, 8.64 | 6.93 ^***^ | 5.99, 7.88 | 0.39 ^***^ | 0.33, 0.47 | 0.55 ^***^ | 0.45, 0.66 | 0.58 ^***^ | 0.48, 0.71 |
| ***Season*** |  |  |  |  |  |  |  |  |  |  |  |  |
| Summer [vs. Spring] | 0.73 | -0.17, 1.62 | 0.46 | -0.42, 1.33 | 0.54 | -0.32, 1.41 | 0.90 | 0.76, 1.08 | 0.92 | 0.77, 1.11 | 0.92 | 0.77, 1.10 |
| Autumn [vs. Spring] | -1.08 ^*^ | -1.93, -0.23 | -0.07 | -0.91, 0.76 | -0.08 | -0.91, 0.75 | 1.16 | 0.99, 1.37 | 1.04 | 0.88, 1.22 | 1.04 | 0.88, 1.23 |
| Winter [vs. Spring] | -1.21 ^**^ | -2.06, -0.36 | -0.08 | -0.91, 0.75 | -0.11 | -0.93, 0.72 | 1.18 ^*^ | 1.01, 1.39 | 1.06 | 0.90, 1.24 | 1.06 | 0.90, 1.25 |
| 18 country intercept variance^‡^ | 12.72 | | 7.87 | | 6.34 | | 0.10 | | 0.07 | | 0.07 | |
| Observations | 16302 | | 16302 | | 16302 | | 16302 | | 16302 | | 16302 | |
| Marginal R^2^ / Conditional R^2^ | 0.151 / 0.179 | | 0.202 / 0.219 | | 0.216 / 0.230 | | 0.173 / 0.196 | | 0.229 / 0.246 | | 0.235 / 0.250 | |

Q = quartile for greenspace and quintile for income; HH = Household; INS = Inclusion of Nature in Self Scale; Married includes cohabiting; Seasons are approximate since ‘Spring’ data were collected in June and referred to the ‘last 4 weeks’ (i.e. May-June), ‘Summer’ in September (i.e. August-September), ‘Autumn’ in December (November to December), and ‘Winter’ in March (i.e. February – March), seasons were reversed for Australia; ^‡^ Variance of country-level intercepts from the random effects component of the model; Marginal R^2^ includes only fixed effects and Conditional R^2^ includes the random country effect, R^2^  for binary outcomes = Nakawaga Pseudo R^2^; ^*^ *p<0.05 ^**^ p<0.01 ^***^ p<0.001.*

**Supplementary Table S3**: Models for medication use outcomes

|  | **Depression medication use** | | | | | | **Anxiety medication use** | | | | | |
| --- | --- | --- | --- | --- | --- | --- | --- | --- | --- | --- | --- | --- |
|  | **Residential + covariates** | | **+ Visits** | | **+ INS** | | **Residential only + covariates** | | **+ Visits** | | **+ INS** | |
|  | *Estimates* | *95% CIs* | *Estimates* | *95% CIs* | *Estimates* | *95% CIs* | *Odds Ratios* | *95% CIs* | *Odds Ratios* | *95% CIs* | *Odds Ratios* | *95% CIs* |
| (Intercept) | 0.07 *** | 0.04, 0.10 | 0.06 *** | 0.04, 0.09 | 0.06 *** | 0.04, 0.09 | 0.06 *** | 0.04, 0.09 | 0.07 *** | 0.05, 0.10 | 0.06 *** | 0.04, 0.09 |
| ***Residential exposure [within 1000m]*** |  |  |  |  |  |  |  |  |  |  |  |  |
| Greenspace [Q2 vs. Q1] | 0.99 | 0.83, 1.19 | 0.99 | 0.82, 1.19 | 0.99 | 0.83, 1.19 | 1.15 | 0.97, 1.37 | 1.15 | 0.96, 1.36 | 1.15 | 0.97, 1.36 |
| Greenspace [Q3 vs. Q1] | 0.84 | 0.70, 1.02 | 0.85 | 0.70, 1.03 | 0.85 | 0.71, 1.03 | 1.04 | 0.87, 1.24 | 1.03 | 0.86, 1.23 | 1.03 | 0.86, 1.24 |
| Greenspace [Q4 vs. Q1] | 0.98 | 0.81, 1.17 | 0.99 | 0.82, 1.19 | 0.99 | 0.82, 1.19 | 1.07 | 0.90, 1.28 | 1.05 | 0.88, 1.25 | 1.05 | 0.88, 1.25 |
| Inland blue [Yes vs. No] | 0.96 | 0.84, 1.11 | 0.96 | 0.83, 1.10 | 0.96 | 0.83, 1.10 | 1.00 | 0.88, 1.15 | 1.00 | 0.87, 1.14 | 1.00 | 0.87, 1.14 |
| Coastal blue [Yes vs. No] | 0.87 | 0.69, 1.11 | 0.90 | 0.71, 1.15 | 0.90 | 0.71, 1.15 | 0.86 | 0.69, 1.08 | 0.82 | 0.65, 1.03 | 0.82 | 0.65, 1.03 |
| ***Visits***  ***[last 4 weeks]*** |  |  |  |  |  |  |  |  |  |  |  |  |
| Green | / | / | 0.99 ** | 0.98, 1.00 | 0.99 * | 0.98, 1.00 | / | / | 1.00 | 0.99, 1.00 | 1.00 | 0.99, 1.01 |
| Green ^2^ | / | / | 1.00 ** | 1.00, 1.00 | 1.00 * | 1.00, 1.00 | / | / | 1.00 | 1.00, 1.00 | 1.00 | 1.00, 1.00 |
| Inland blue | / | / | 1.01 | 0.99, 1.02 | 1.01 | 0.99, 1.02 | / | / | 1.02 * | 1.00, 1.03 | 1.02 * | 1.00, 1.03 |
| Inland blue ^2^ | / | / | 1.00 | 1.00, 1.00 | 1.00 | 1.00, 1.00 | / | / | 1.00 | 1.00, 1.00 | 1.00 | 1.00, 1.00 |
| Coastal blue | / | / | 0.99 | 0.98, 1.01 | 0.99 | 0.98, 1.01 | / | / | 1.01 | 1.00, 1.02 | 1.01 | 1.00, 1.02 |
| Coastal blue ^2^ | / | / | 1.00 | 1.00, 1.00 | 1.00 | 1.00, 1.00 | / | / | 1.00 | 1.00, 1.00 | 1.00 | 1.00, 1.00 |
| ***Nature Connectedness*** |  |  |  |  |  |  |  |  |  |  |  |  |
| INS | / | / | / | / | 0.83 * | 0.70, 1.00 | / | / | / | / | 0.96 | 0.81, 1.14 |
| INS^2^ | / | / | / | / | 1.02 | 1.00, 1.04 | / | / | / | / | 1.00 | 0.98, 1.02 |
| ***Socio-demographics*** |  |  |  |  |  |  |  |  |  |  |  |  |
| Sex [Male vs. Female] | 0.86 * | 0.76, 0.98 | 0.87 * | 0.76, 0.99 | 0.87 * | 0.76, 0.99 | 0.85 ** | 0.75, 0.96 | 0.84 ** | 0.74, 0.95 | 0.83 ** | 0.73, 0.94 |
| Age [30-39 vs. 18-29] | 1.08 | 0.86, 1.36 | 1.07 | 0.85, 1.35 | 1.08 | 0.85, 1.36 | 0.86 | 0.70, 1.06 | 0.88 | 0.72, 1.08 | 0.88 | 0.72, 1.09 |
| Age [40-49 vs. 18-29] | 1.35 ** | 1.08, 1.68 | 1.33 * | 1.06, 1.67 | 1.34 * | 1.07, 1.68 | 0.89 | 0.73, 1.10 | 0.92 | 0.75, 1.13 | 0.93 | 0.76, 1.15 |
| Age [50-59 vs. 18-29] | 1.25 * | 1.00, 1.57 | 1.25 | 0.99, 1.57 | 1.26 * | 1.01, 1.59 | 0.82 | 0.67, 1.02 | 0.86 | 0.70, 1.06 | 0.87 | 0.71, 1.08 |
| Age [60+ vs. 18-29] | 0.79 | 0.60, 1.04 | 0.79 | 0.60, 1.04 | 0.80 | 0.61, 1.06 | 0.50 *** | 0.38, 0.64 | 0.52 *** | 0.40, 0.67 | 0.52 *** | 0.40, 0.68 |
| HH income [Q2 vs. Q1] | 0.85 | 0.69, 1.05 | 0.85 | 0.69, 1.05 | 0.86 | 0.70, 1.06 | 0.93 | 0.76, 1.14 | 0.93 | 0.76, 1.13 | 0.93 | 0.76, 1.14 |
| HH income [Q3 vs. Q1] | 0.74 ** | 0.59, 0.93 | 0.74 ** | 0.59, 0.93 | 0.74 * | 0.59, 0.93 | 0.88 | 0.71, 1.08 | 0.88 | 0.71, 1.09 | 0.87 | 0.70, 1.08 |
| HH income [Q4 vs. Q1] | 0.79 | 0.63, 1.00 | 0.79 | 0.63, 1.00 | 0.80 | 0.63, 1.01 | 0.86 | 0.69, 1.07 | 0.86 | 0.69, 1.07 | 0.86 | 0.69, 1.07 |
| HH income [Q5 vs. Q1] | 0.73 * | 0.57, 0.93 | 0.74 * | 0.58, 0.94 | 0.74 * | 0.58, 0.94 | 0.78 * | 0.62, 0.98 | 0.77 * | 0.61, 0.97 | 0.77 * | 0.61, 0.96 |
| HH income [prefer not answer vs. Q1] | 0.52 *** | 0.40, 0.66 | 0.51 *** | 0.40, 0.66 | 0.52 *** | 0.40, 0.66 | 0.69 ** | 0.54, 0.88 | 0.69 ** | 0.55, 0.88 | 0.70 ** | 0.55, 0.88 |
| Retired [vs. Unemployed] | 0.75 * | 0.58, 0.96 | 0.75 * | 0.59, 0.96 | 0.75 * | 0.58, 0.96 | 0.98 | 0.76, 1.26 | 0.97 | 0.75, 1.25 | 0.97 | 0.75, 1.25 |
| Home maker [vs. Unemployed] | 0.60 ** | 0.43, 0.82 | 0.60 ** | 0.43, 0.83 | 0.60 ** | 0.43, 0.83 | 0.91 | 0.68, 1.21 | 0.93 | 0.69, 1.24 | 0.93 | 0.69, 1.24 |
| In education [vs. Unemployed] | 0.61 ** | 0.44, 0.84 | 0.61 ** | 0.44, 0.84 | 0.61 ** | 0.44, 0.84 | 0.80 | 0.60, 1.07 | 0.78 | 0.59, 1.05 | 0.78 | 0.58, 1.05 |
| Employed [vs. Unemployed] | 0.67 *** | 0.56, 0.81 | 0.67 *** | 0.56, 0.80 | 0.67 *** | 0.56, 0.80 | 0.86 | 0.72, 1.02 | 0.85 | 0.71, 1.01 | 0.84 | 0.71, 1.00 |
| University ed.[ Yes vs. No] | 1.08 | 0.94, 1.24 | 1.08 | 0.94, 1.24 | 1.09 | 0.95, 1.25 | 0.93 | 0.82, 1.06 | 0.93 | 0.82, 1.06 | 0.93 | 0.82, 1.06 |
| Ill/disability [Yes vs. No] | 5.01 *** | 4.31, 5.82 | 5.00 *** | 4.30, 5.82 | 4.99 *** | 4.29, 5.80 | 3.19 *** | 2.78, 3.65 | 3.12 *** | 2.72, 3.57 | 3.12 *** | 2.72, 3.58 |
| Married [Yes vs. No] | 0.96 | 0.80, 1.14 | 0.96 | 0.80, 1.14 | 0.96 | 0.81, 1.15 | 0.99 | 0.84, 1.17 | 0.97 | 0.83, 1.15 | 0.98 | 0.83, 1.16 |
| Adults in HH [2 vs. 1] | 0.72 ** | 0.60, 0.88 | 0.73 ** | 0.60, 0.88 | 0.73 ** | 0.60, 0.88 | 0.97 | 0.81, 1.17 | 0.99 | 0.82, 1.19 | 0.99 | 0.82, 1.19 |
| Adults in HH [3+ vs. 1] | 0.99 | 0.81, 1.21 | 0.99 | 0.81, 1.22 | 0.99 | 0.81, 1.21 | 0.86 | 0.70, 1.04 | 0.88 | 0.73, 1.07 | 0.87 | 0.72, 1.06 |
| Children in HH [1 vs. 0] | 1.02 | 0.84, 1.24 | 1.02 | 0.84, 1.24 | 1.01 | 0.83, 1.24 | 1.13 | 0.94, 1.35 | 1.10 | 0.92, 1.32 | 1.11 | 0.92, 1.32 |
| Children in HH[2+ vs. 0] | 0.99 | 0.80, 1.23 | 1.01 | 0.81, 1.26 | 1.01 | 0.81, 1.26 | 1.11 | 0.92, 1.36 | 1.10 | 0.91, 1.35 | 1.10 | 0.90, 1.34 |
| Owns dog [Yes vs. No] | 1.23 ** | 1.07, 1.42 | 1.27 ** | 1.10, 1.46 | 1.27 ** | 1.10, 1.47 | 1.17 * | 1.03, 1.34 | 1.16 * | 1.01, 1.33 | 1.17 * | 1.02, 1.34 |
| Owns car [Yes vs. No] | 0.89 | 0.75, 1.05 | 0.91 | 0.76, 1.08 | 0.92 | 0.77, 1.09 | 0.89 | 0.75, 1.05 | 0.89 | 0.75, 1.05 | 0.89 | 0.75, 1.05 |
| Days active [1-4 vs. 0] | 0.72 *** | 0.62, 0.83 | 0.75 *** | 0.64, 0.87 | 0.76 *** | 0.65, 0.89 | 1.13 | 0.98, 1.31 | 1.08 | 0.93, 1.25 | 1.10 | 0.94, 1.27 |
| Days active [5+ vs. 0] | 0.68 *** | 0.56, 0.82 | 0.72 ** | 0.59, 0.89 | 0.74 ** | 0.60, 0.91 | 1.02 | 0.84, 1.23 | 0.97 | 0.80, 1.18 | 0.98 | 0.80, 1.19 |
| ***Season*** |  |  |  |  |  |  |  |  |  |  |  |  |
| Summer [vs. Spring] | 0.95 | 0.78, 1.14 | 0.96 | 0.79, 1.15 | 0.95 | 0.79, 1.14 | 1.09 | 0.92, 1.30 | 1.09 | 0.91, 1.30 | 1.09 | 0.91, 1.30 |
| Autumn [vs. Spring] | 0.96 | 0.80, 1.14 | 0.94 | 0.79, 1.13 | 0.94 | 0.79, 1.12 | 0.99 | 0.84, 1.18 | 1.02 | 0.86, 1.21 | 1.02 | 0.86, 1.21 |
| Winter [vs. Spring] | 0.95 | 0.80, 1.13 | 0.94 | 0.78, 1.12 | 0.93 | 0.78, 1.12 | 1.07 | 0.90, 1.26 | 1.10 | 0.93, 1.30 | 1.10 | 0.93, 1.30 |
| ***Medication use*** |  |  |  |  |  |  |  |  |  |  |  |  |
| Anxiety | 8.82 *** | 7.65, 10.17 | 8.86 *** | 7.69, 10.22 | 8.84 *** | 7.67, 10.20 | / | / | / | / | / | / |
| Depression | / | / | / | / | / | / | 8.83 *** | 7.66, 10.17 | 8.75 *** | 7.60, 10.08 | 8.83 *** | 7.66, 10.17 |
| 18 country intercept variance^‡^ | 0.20 | | 0.18 | | 0.18 | | 0.14 | | 0.13 | | 0.13 | |
| Observations | 16302 | | 16302 | | 16302 | | 16302 | | 16302 | | 16302 | |
| Marginal R^2^ / Conditional R^2^ | 0.310 / 0.350 | | 0.315 / 0.351 | | 0.315 / 0.351 | | 0.238 / 0.269 | | 0.238 / 0.266 | | 0.240 / 0.269 | |

Q = quartile for greenspace and quintile for income; HH = Household; INS = Inclusion of Nature in Self Scale; Married includes cohabiting; Seasons are approximate since ‘Spring’ data were collected in June and referred to the ‘last 4 weeks’ (i.e. May-June), ‘Summer’ in September (i.e. August-September), ‘Autumn’ in December (November to December), and ‘Winter’ in March (i.e. February – March), seasons were reversed for Australia; ^‡^ Variance of country-level intercepts from the random effects component of the model; Marginal R^2^ includes only fixed effects and Conditional R^2^ includes the random country effect, R^2^  for binary outcomes = Nakawaga Pseudo R^2^; *^*^ p<0.05 ^**^ p<0.01  ^***^ p<0.001.*

**Supplementary Table S4***:* WHO-5 scores for each seasonal wave of data collection

|  | **WHO-5 (0-100)**  **Spring** | | **WHO-5 (0-100) Summer** | | **WHO-5 (0-100) Autumn** | | **WHO-5 (0-100)**  **Winter** | |
| --- | --- | --- | --- | --- | --- | --- | --- | --- |
| *Predictors* | *Estimates* | *CI* | *Estimates* | *CI* | *Estimates* | *CI* | *Estimates* | *CI* |
| (Intercept) | 46.83 ^***^ | 43.39, 50.26 | 47.45 ^***^ | 43.86, 51.05 | 47.54 ^***^ | 44.00, 51.07 | 50.72 ^***^ | 47.20, 54.24 |
| ***Residential exposure [within 1000m]*** |  |  |  |  |  |  |  |  |
| Greenspace [Q2 vs. Q1] | 1.44 | -0.24, 3.13 | 0.84 | -0.91, 2.60 | -0.40 | -2.04, 1.24 | 0.18 | -1.47, 1.82 |
| Greenspace [Q3 vs. Q1] | 1.78 ^*^ | 0.07, 3.49 | 1.15 | -0.64, 2.95 | 0.39 | -1.32, 2.10 | 1.09 | -0.57, 2.74 |
| Greenspace [Q4 vs. Q1] | 1.33 | -0.44, 3.10 | 1.01 | -0.83, 2.84 | -0.51 | -2.22, 1.20 | -0.53 | -2.24, 1.18 |
| Inland blue [Yes vs. No] | 0.56 | -0.72, 1.85 | 0.10 | -1.26, 1.46 | 0.37 | -0.92, 1.67 | -0.84 | -2.12, 0.43 |
| Coastal blue [Yes vs. No] | 0.37 | -1.68, 2.41 | 0.36 | -1.76, 2.47 | -0.07 | -2.17, 2.03 | 1.71 | -0.37, 3.79 |
| ***Visits [last 4 weeks]*** |  |  |  |  |  |  |  |  |
| Green | 0.24 *** | 0.15, 0.33 | 0.22 *** | 0.13, 0.31 | 0.28 *** | 0.20, 0.37 | 0.31 *** | 0.22, 0.40 |
| Green ^2^ | -0.00 | -0.00, 0.00 | -0.00 * | -0.01, -0.00 | 0.00 | -0.00, 0.00 | -0.00 *** | -0.01, -0.00 |
| Inland blue | 0.09 | -0.04, 0.23 | 0.14 * | 0.00, 0.29 | 0.08 | -0.06, 0.23 | 0.14 * | 0.01, 0.28 |
| Inland blue ^2^ | -0.00 | -0.01, 0.00 | 0.00 | -0.00, 0.01 | -0.00 * | -0.01, -0.00 | -0.00 | -0.01, 0.00 |
| Coastal blue | 0.12 | -0.01, 0.25 | 0.23 *** | 0.10, 0.36 | 0.21 ** | 0.07, 0.35 | 0.20 ** | 0.06, 0.34 |
| Coastal blue ^2^ | 0.00 | -0.00, 0.00 | -0.00 | -0.00, 0.00 | -0.00 | -0.01, 0.00 | -0.00 | -0.01, 0.00 |
| ***Nature Connectedness*** |  |  |  |  |  |  |  |  |
| INS | 0.85 | -1.02, 2.73 | 2.41 * | 0.51, 4.31 | 2.29 * | 0.54, 4.03 | 3.18 *** | 1.46, 4.90 |
| INS^2^ | 0.05 | -0.16, 0.27 | -0.09 | -0.32, 0.13 | -0.14 | -0.35, 0.06 | -0.11 | -0.31, 0.09 |
| ***Socio-demographics*** |  |  |  |  |  |  |  |  |
| Sex [Male vs. Female] | 0.11 | -1.11, 1.33 | 0.13 | -1.14, 1.40 | 1.63 ^**^ | 0.43, 2.82 | -0.41 | -1.59, 0.77 |
| Age [30-39 vs. 18-29] | -0.81 | -2.97, 1.34 | 1.88 | -0.37, 4.13 | 0.53 | -1.58, 2.64 | -0.90 | -2.98, 1.19 |
| Age [40-49 vs. 18-29] | 1.10 | -1.05, 3.26 | 3.95 ^***^ | 1.68, 6.22 | 1.49 | -0.63, 3.61 | 0.40 | -1.70, 2.50 |
| Age [50-59 vs. 18-29] | 3.14 ^**^ | 0.95, 5.33 | 3.71 ^**^ | 1.43, 6.00 | 3.34 ^**^ | 1.19, 5.48 | 0.10 | -2.03, 2.23 |
| Age [60+ vs. 18-29] | 4.89 ^***^ | 2.36, 7.42 | 6.23 ^***^ | 3.59, 8.87 | 5.44 ^***^ | 3.02, 7.87 | 4.25 ^***^ | 1.89, 6.60 |
| HH income [Q2 vs. Q1] | 1.46 | -0.73, 3.64 | 0.63 | -1.65, 2.90 | 1.12 | -1.05, 3.29 | 2.36 ^*^ | 0.21, 4.52 |
| HH income [Q3 vs. Q1] | 3.56 ^**^ | 1.34, 5.79 | 2.70 ^*^ | 0.38, 5.02 | 0.98 | -1.25, 3.20 | 2.90 ^**^ | 0.73, 5.07 |
| HH income [Q4 vs. Q1] | 3.72 ^**^ | 1.44, 5.99 | 2.00 | -0.33, 4.32 | 2.57 ^*^ | 0.34, 4.81 | 3.29 ^**^ | 1.06, 5.52 |
| HH income [Q5 vs. Q1] | 5.45 ^***^ | 3.21, 7.70 | 5.45 ^***^ | 3.07, 7.83 | 3.26 ^**^ | 0.97, 5.54 | 4.74 ^***^ | 2.51, 6.96 |
| HH income [prefer not answer vs. Q1] | 3.10 ^**^ | 0.78, 5.41 | 2.54 ^*^ | 0.16, 4.92 | 2.50 ^*^ | 0.24, 4.76 | 2.07 | -0.15, 4.29 |
| Retired [vs. Unemployed] | 6.82 ^***^ | 4.25, 9.38 | 6.50 ^***^ | 3.80, 9.19 | 4.77 ^***^ | 2.23, 7.31 | 5.62 ^***^ | 3.16, 8.07 |
| Home maker [vs. Unemployed] | 3.90 ^*^ | 0.80, 6.99 | 3.46 ^*^ | 0.21, 6.72 | 3.17 ^*^ | 0.08, 6.26 | 1.03 | -1.92, 3.98 |
| In education [vs. Unemployed] | 5.22 ^***^ | 2.26, 8.17 | 6.80 ^***^ | 3.63, 9.97 | 4.26 ^**^ | 1.30, 7.22 | 2.93 | -0.08, 5.95 |
| Employed [vs. Unemployed] | 4.86 ^***^ | 2.98, 6.73 | 3.92 ^***^ | 1.93, 5.91 | 4.55 ^***^ | 2.66, 6.44 | 3.54 ^***^ | 1.71, 5.38 |
| University ed.[ Yes vs. No] | -0.66 | -1.92, 0.61 | 0.76 | -0.57, 2.08 | -0.20 | -1.44, 1.04 | 0.49 | -0.75, 1.72 |
| Ill/disability [Yes vs. No] | -9.99 ^***^ | -11.27, -8.70 | -9.57 ^***^ | -10.92, -8.22 | -10.23 ^***^ | -11.50, -8.97 | -9.70 ^***^ | -10.97, -8.43 |
| Married [Yes vs. No] | -0.12 | -1.84, 1.61 | 0.54 | -1.20, 2.27 | 2.19 ^**^ | 0.58, 3.81 | 1.54 | -0.13, 3.21 |
| Adults in HH [2 vs. 1] | 0.09 | -1.86, 2.04 | 0.98 | -1.01, 2.97 | -1.02 | -2.90, 0.86 | 0.65 | -1.24, 2.53 |
| Adults in HH [3+ vs. 1] | -0.11 | -2.09, 1.86 | -0.88 | -2.93, 1.18 | -1.87 | -3.85, 0.11 | 0.68 | -1.28, 2.63 |
| Children in HH [1 vs. 0] | -0.31 | -2.12, 1.50 | 0.42 | -1.44, 2.27 | -1.02 | -2.82, 0.77 | 0.46 | -1.32, 2.25 |
| Children in HH[2+ vs. 0] | -0.52 | -2.53, 1.49 | -1.03 | -3.20, 1.13 | -0.24 | -2.17, 1.69 | 0.37 | -1.60, 2.34 |
| Owns dog [Yes vs. No] | -1.12 | -2.50, 0.26 | -0.24 | -1.66, 1.17 | 0.66 | -0.69, 2.01 | -1.26 | -2.59, 0.07 |
| Owns car [Yes vs. No] | 3.44 ^***^ | 1.72, 5.16 | 2.44 ^**^ | 0.61, 4.27 | 3.62 ^***^ | 1.91, 5.33 | 2.53 ^**^ | 0.84, 4.21 |
| Days active [1-4 vs. 0] | 4.89 ^***^ | 3.37, 6.41 | 3.77 ^***^ | 2.20, 5.33 | 5.63 ^***^ | 4.20, 7.05 | 4.37 ^***^ | 2.93, 5.81 |
| Days active [5+ vs. 0] | 6.82 ^***^ | 4.94, 8.69 | 5.18 ^***^ | 3.21, 7.14 | 8.11 ^***^ | 6.24, 9.98 | 6.76 ^***^ | 4.91, 8.60 |
| **Random Effects** | | | | | | | | |
| 18 country intercept variance^‡^ | 2.86 | | 3.89 | | 7.81 | | 8.98 | |
| Observations | 4122 | | 3546 | | 4248 | | 4386 | |
| Marginal R^2^ / Conditional R^2^ | 0.205 / 0.211 | | 0.243 / 0.252 | | 0.222 / 0.239 | | 0.227 / 0.246 | |

Q = quartile for greenspace and quintile for income; HH = Household; INS = Inclusion of Nature in Self Scale; Married includes cohabiting; Seasons are approximate since ‘Spring’ data were collected in June and referred to the ‘last 4 weeks’ (i.e. May-June), ‘Summer’ in September (i.e. August-September), ‘Autumn’ in December (November to December), and ‘Winter’ in March (i.e. February – March), seasons were reversed for Australia; ^‡^ Variance of country level intercepts from the random effects component of the model; Marginal R^2^ includes only fixed effects and Conditional R^2^ includes the random country effect; *^*^ p<0.05  ^**^ p<0.01   ^***^ p<0.001.*

**Supplementary Table S5***:* WHO-5 scores for each country separately

|  | **Australia (Queensland)** | | **Bulgaria** | | **USA (California)** | | **Canada** | |
| --- | --- | --- | --- | --- | --- | --- | --- | --- |
| *Predictors* | *Estimates* | *CI* | *Estimates* | *CI* | *Estimates* | *CI* | *Estimates* | *CI* |
| (Intercept) | 46.46 *** | 38.24, 54.68 | 48.35 *** | 39.76, 56.94 | 50.00 *** | 41.84, 58.17 | 49.44 *** | 41.48, 57.40 |
| Greenspace [Q2 vs. Q1] | 2.74 | -1.18, 6.65 | 0.22 | -3.34, 3.78 | 0.53 | -2.87, 3.93 | 1.36 | -2.29, 5.02 |
| Greenspace [Q3 vs. Q1] | 3.03 | -1.23, 7.29 | 2.16 | -2.13, 6.45 | -0.27 | -4.58, 4.04 | -0.06 | -3.84, 3.71 |
| Greenspace [Q4 vs. Q1] | 2.61 | -0.95, 6.17 | -1.57 | -5.30, 2.15 | -0.07 | -3.35, 3.22 | -1.22 | -5.08, 2.65 |
| Inland blue [Yes vs. No] | 0.14 | -2.65, 2.93 | 0.61 | -2.82, 4.04 | -1.79 | -4.98, 1.39 | 2.04 | -0.84, 4.91 |
| Coastal blue [Yes vs. No] | -0.06 | -4.63, 4.52 | -2.16 | -10.49, 6.17 | 1.21 | -5.89, 8.32 | 0.43 | -6.66, 7.52 |
| Green | 0.41 *** | 0.20, 0.62 | 0.48 *** | 0.28, 0.68 | 0.42 *** | 0.21, 0.64 | 0.13 | -0.09, 0.35 |
| Green ^2^ | -0.01 ** | -0.02, -0.00 | -0.01 * | -0.01, -0.00 | -0.00 | -0.01, 0.00 | -0.00 | -0.01, 0.00 |
| Inland blue | 0.09 | -0.25, 0.43 | 0.14 | -0.17, 0.46 | -0.19 | -0.53, 0.15 | 0.20 | -0.13, 0.52 |
| Inland blue ^2^ | 0.01 | -0.01, 0.02 | -0.01 | -0.02, 0.01 | 0.00 | -0.01, 0.01 | -0.00 | -0.01, 0.01 |
| Coastal blue | 0.01 | -0.31, 0.33 | 0.28 | -0.03, 0.60 | 0.19 | -0.12, 0.51 | 0.19 | -0.17, 0.54 |
| Coastal blue ^2^ | 0.01 | -0.00, 0.02 | -0.01 | -0.02, 0.00 | -0.00 | -0.01, 0.01 | -0.00 | -0.02, 0.01 |
| INS | 2.27 | -1.45, 6.00 | -0.11 | -4.76, 4.53 | 3.48 | -0.36, 7.31 | 4.30 * | 0.52, 8.07 |
| INS^2^ | -0.16 | -0.62, 0.29 | 0.22 | -0.30, 0.74 | -0.23 | -0.69, 0.24 | -0.30 | -0.76, 0.16 |
| Sex [Male vs. Female] | 0.91 | -1.92, 3.74 | 1.14 | -1.73, 4.02 | -1.00 | -3.69, 1.68 | -0.79 | -3.48, 1.91 |
| Age [30-39 vs. 18-29] | 2.75 | -2.01, 7.51 | 1.30 | -3.53, 6.13 | -1.03 | -5.54, 3.49 | -1.98 | -6.58, 2.63 |
| Age [40-49 vs. 18-29] | 0.10 | -4.65, 4.85 | 2.98 | -1.86, 7.81 | 0.87 | -3.58, 5.33 | -2.63 | -7.42, 2.16 |
| Age [50-59 vs. 18-29] | 3.61 | -1.24, 8.47 | 2.95 | -1.90, 7.80 | 0.04 | -4.70, 4.78 | 2.29 | -2.37, 6.96 |
| Age [60+ vs. 18-29] | 7.62 ** | 2.15, 13.10 | 3.82 | -1.72, 9.36 | 3.51 | -2.06, 9.09 | 0.79 | -4.44, 6.01 |
| HH income [Q2 vs. Q1] | -1.68 | -6.80, 3.44 | -0.14 | -8.25, 7.97 | 5.41 | -0.62, 11.43 | 6.53 * | 1.11, 11.96 |
| HH income [Q3 vs. Q1] | -1.64 | -7.38, 4.11 | 6.13 | -1.45, 13.70 | 7.39 * | 1.60, 13.19 | 4.10 | -1.12, 9.32 |
| HH income [Q4 vs. Q1] | -0.04 | -5.64, 5.56 | 2.63 | -3.83, 9.09 | 5.03 | -0.48, 10.54 | 5.24 * | 0.14, 10.34 |
| HH income [Q5 vs. Q1] | 0.58 | -5.48, 6.63 | 5.16 | -1.02, 11.35 | 10.15 *** | 5.10, 15.21 | 6.38 * | 1.38, 11.39 |
| HH income [prefer not answer vs. Q1] | 1.72 | -3.91, 7.35 | 5.33 | -1.67, 12.34 | 6.00 * | 0.39, 11.61 | 3.50 | -2.93, 9.92 |
| Retired [vs. Unemployed] | 7.44 ** | 2.32, 12.56 | 1.26 | -4.74, 7.27 | 6.05 * | 0.64, 11.47 | 5.09 | -0.59, 10.77 |
| Home maker [vs. Unemployed] | 0.36 | -5.15, 5.88 | 1.60 | -3.24, 6.44 | 2.46 | -3.51, 8.42 | -2.60 | -10.37, 5.16 |
| In education [vs. Unemployed] | 7.14 * | 0.46, 13.83 | 5.32 | -0.45, 11.08 | -3.48 | -9.69, 2.73 | 1.20 | -7.23, 9.64 |
| Employed [vs. Unemployed] | 4.52 * | 0.62, 8.43 | -0.51 | -4.11, 3.10 | 1.25 | -2.61, 5.11 | 2.23 | -2.29, 6.76 |
| University ed.[ Yes vs. No] | -0.78 | -3.52, 1.96 | 1.32 | -1.87, 4.51 | -0.51 | -3.40, 2.37 | 0.50 | -2.22, 3.21 |
| Ill/disability [Yes vs. No] | -10.40 *** | -13.23, -7.58 | -12.33 *** | -15.85, -8.81 | -11.19 *** | -13.95, -8.44 | -8.43 *** | -11.18, -5.69 |
| Married [Yes vs. No] | 2.80 | -0.76, 6.37 | -3.12 | -6.66, 0.42 | 3.42 * | 0.07, 6.77 | 5.55 ** | 1.59, 9.51 |
| Adults in HH [2 vs. 1] | 2.56 | -1.51, 6.63 | 5.00 * | 0.24, 9.76 | -1.41 | -5.14, 2.33 | -4.97 * | -9.08, -0.86 |
| Adults in HH [3+ vs. 1] | -0.24 | -4.58, 4.10 | 2.15 | -2.71, 7.01 | -0.97 | -4.79, 2.85 | -8.90 *** | -13.34, -4.46 |
| Children in HH [1 vs. 0] | -0.85 | -4.91, 3.21 | 1.42 | -2.35, 5.18 | 1.00 | -3.23, 5.22 | -2.83 | -7.20, 1.54 |
| Children in HH[2+ vs. 0] | -0.55 | -4.91, 3.82 | -3.12 | -7.88, 1.64 | -5.63 * | -10.06, -1.20 | 1.29 | -3.64, 6.22 |
| Owns dog [Yes vs. No] | 0.93 | -1.97, 3.83 | -1.33 | -4.59, 1.94 | -1.43 | -4.11, 1.26 | 0.67 | -2.31, 3.65 |
| Owns car [Yes vs. No] | 3.65 | -1.12, 8.41 | 1.90 | -1.93, 5.72 | 0.28 | -4.05, 4.61 | 6.76 ** | 2.56, 10.96 |
| Days active [1-4 vs. 0] | 3.09 | -0.18, 6.37 | 5.54 ** | 1.97, 9.11 | 4.35 * | 0.96, 7.75 | 5.06 ** | 1.49, 8.62 |
| Days active [5+ vs. 0] | 7.81 *** | 3.68, 11.94 | 7.77 *** | 3.60, 11.93 | 7.01 *** | 3.04, 10.97 | 5.31 * | 0.90, 9.72 |
| Summer | -1.45 | -5.00, 2.10 | 0.60 | -3.37, 4.57 | 1.72 | -2.08, 5.51 | 0.03 | -3.78, 3.84 |
| Autumn | -0.46 | -4.14, 3.21 | -0.72 | -4.47, 3.04 | -0.10 | -3.86, 3.66 | 1.07 | -2.45, 4.58 |
| Winter | -2.52 | -6.20, 1.15 | 3.07 | -0.62, 6.76 | -1.38 | -5.08, 2.32 | 1.10 | -2.43, 4.64 |
| Observations | 842 | | 974 | | 914 | | 885 | |
| R^2^ adj | 0.311 / 0.276 | | 0.216 / 0.181 | | 0.281 / 0.247 | | 0.240 / 0.203 | |

**Supplementary Table S5 continued***:* WHO-5 scores for each country separately

|  | **Czech Republic** | | **Estonia** | | **Finland** | | **France** | |
| --- | --- | --- | --- | --- | --- | --- | --- | --- |
| *Predictors* | *Estimates* | *CI* | *Estimates* | *CI* | *Estimates* | *CI* | *Estimates* | *CI* |
| (Intercept) | 48.43 *** | 39.66, 57.19 | 47.03 *** | 40.23, 53.83 | 48.15 *** | 41.95, 54.35 | 55.04 *** | 46.88, 63.20 |
| Greenspace [Q2 vs. Q1] | -0.37 | -4.86, 4.13 | 0.13 | -3.46, 3.71 | -0.73 | -3.81, 2.35 | -0.45 | -4.50, 3.60 |
| Greenspace [Q3 vs. Q1] | -1.83 | -5.91, 2.25 | 1.43 | -2.31, 5.18 | -4.20 * | -7.66, -0.74 | 1.27 | -2.56, 5.10 |
| Greenspace [Q4 vs. Q1] | -1.91 | -5.94, 2.13 | 0.11 | -3.55, 3.78 | -1.61 | -5.37, 2.15 | 0.78 | -2.88, 4.45 |
| Inland blue [Yes vs. No] | 0.95 | -1.75, 3.66 | -0.99 | -3.68, 1.71 | 3.53 ** | 1.12, 5.95 | -0.03 | -2.72, 2.67 |
| Coastal blue [Yes vs. No] | / | / | 0.07 | -4.16, 4.30 | -1.16 | -4.24, 1.92 | 5.97 | -0.73, 12.67 |
| Green | 0.37 ^***^ | 0.18, 0.55 | 0.23 ^*^ | 0.04, 0.41 | 0.19 ^*^ | 0.03, 0.36 | 0.15 | -0.06, 0.36 |
| Green ^2^ | 0.00 | -0.01, 0.01 | -0.00 | -0.01, 0.00 | -0.00 | -0.01, 0.01 | 0.00 | -0.01, 0.01 |
| Inland blue | -0.02 | -0.31, 0.26 | 0.09 | -0.21, 0.38 | 0.17 | -0.08, 0.43 | 0.21 | -0.13, 0.55 |
| Inland blue ^2^ | -0.00 | -0.01, 0.01 | 0.00 | -0.01, 0.02 | -0.00 | -0.01, 0.01 | -0.00 | -0.02, 0.01 |
| Coastal blue | 0.18 | -0.27, 0.63 | 0.05 | -0.30, 0.41 | 0.15 | -0.13, 0.42 | 0.57 ^**^ | 0.23, 0.92 |
| Coastal blue ^2^ | -0.00 | -0.02, 0.01 | 0.00 | -0.01, 0.02 | 0.00 | -0.01, 0.01 | -0.01 ^*^ | -0.03, -0.00 |
| INS | 5.41 ^**^ | 1.36, 9.46 | -0.74 | -4.70, 3.23 | 1.26 | -2.36, 4.89 | -1.86 | -6.00, 2.28 |
| INS^2^ | -0.40 | -0.86, 0.06 | 0.21 | -0.24, 0.66 | 0.04 | -0.38, 0.46 | 0.39 | -0.08, 0.86 |
| Sex [Male vs. Female] | -0.23 | -2.89, 2.43 | -2.49 | -5.10, 0.11 | 1.68 | -0.60, 3.95 | 1.15 | -1.68, 3.98 |
| Age [30-39 vs. 18-29] | 3.69 | -1.03, 8.40 | -2.93 | -7.41, 1.55 | -1.71 | -5.84, 2.43 | -3.95 | -9.32, 1.41 |
| Age [40-49 vs. 18-29] | 4.21 | -0.68, 9.10 | 2.59 | -1.79, 6.97 | 0.34 | -3.97, 4.65 | 3.08 | -1.98, 8.15 |
| Age [50-59 vs. 18-29] | 3.63 | -1.31, 8.57 | 0.31 | -4.18, 4.79 | 0.05 | -4.22, 4.31 | 1.07 | -3.96, 6.11 |
| Age [60+ vs. 18-29] | 5.83 ^*^ | 0.25, 11.40 | 3.04 | -1.78, 7.86 | 5.86 ^*^ | 1.06, 10.66 | -0.30 | -6.21, 5.61 |
| HH income [Q2 vs. Q1] | 3.76 | -0.73, 8.25 | 3.12 | -0.85, 7.08 | 3.38 | -0.43, 7.20 | -1.13 | -5.57, 3.30 |
| HH income [Q3 vs. Q1] | -1.66 | -6.21, 2.90 | 4.01 | -0.18, 8.21 | 5.42 ^*^ | 1.18, 9.67 | 4.20 | -0.46, 8.85 |
| HH income [Q4 vs. Q1] | 1.02 | -3.68, 5.72 | 5.52 ^*^ | 1.09, 9.95 | 4.09 | -0.36, 8.55 | 0.19 | -4.80, 5.18 |
| HH income [Q5 vs. Q1] | 5.20 | -0.01, 10.42 | 5.91 ^*^ | 0.63, 11.18 | 8.43 ^***^ | 3.43, 13.44 | 3.96 | -1.47, 9.39 |
| HH income [prefer not answer vs. Q1] | 1.54 | -3.27, 6.34 | 4.21 ^*^ | 0.18, 8.25 | 6.08 ^**^ | 1.69, 10.48 | 0.81 | -3.91, 5.54 |
| Retired [vs. Unemployed] | 6.40 ^*^ | 0.29, 12.51 | 6.17 ^*^ | 0.27, 12.06 | 4.17 ^*^ | 0.08, 8.26 | 6.43 ^*^ | 0.81, 12.04 |
| Home maker [vs. Unemployed] | 4.27 | -3.23, 11.78 | 0.79 | -7.46, 9.03 | 0.38 | -7.51, 8.27 | 8.82 ^*^ | 1.40, 16.24 |
| In education [vs. Unemployed] | -0.38 | -8.18, 7.41 | 5.57 | -1.14, 12.29 | 3.76 | -1.07, 8.60 | 5.67 | -1.27, 12.61 |
| Employed [vs. Unemployed] | 4.32 | -0.75, 9.38 | 5.57 ^**^ | 1.50, 9.64 | 2.70 | -0.77, 6.16 | 6.20 ^**^ | 1.91, 10.50 |
| University ed.[ Yes vs. No] | -0.02 | -2.93, 2.89 | -0.02 | -2.70, 2.67 | 0.05 | -2.31, 2.42 | -0.59 | -3.46, 2.29 |
| Ill/disability [Yes vs. No] | -6.96 ^***^ | -9.66, -4.27 | -8.31 ^***^ | -11.03, -5.59 | -7.75 ^***^ | -10.08, -5.41 | -10.95 ^***^ | -13.85, -8.04 |
| Married [Yes vs. No] | -0.49 | -3.79, 2.80 | 1.48 | -1.97, 4.93 | 0.24 | -3.74, 4.23 | 3.84 | -0.14, 7.81 |
| Adults in HH [2 vs. 1] | 3.12 | -1.06, 7.31 | -1.02 | -4.80, 2.77 | -0.49 | -4.64, 3.65 | -4.59 ^*^ | -9.05, -0.12 |
| Adults in HH [3+ vs. 1] | 2.29 | -2.32, 6.90 | -0.72 | -4.83, 3.40 | 1.04 | -3.72, 5.80 | -6.32 ^**^ | -11.12, -1.53 |
| Children in HH [1 vs. 0] | 2.11 | -1.69, 5.91 | 2.29 | -1.37, 5.96 | -1.49 | -5.66, 2.68 | 0.31 | -3.98, 4.60 |
| Children in HH[2+ vs. 0] | -0.23 | -4.37, 3.91 | 2.81 | -1.20, 6.82 | -0.02 | -3.91, 3.88 | 2.33 | -2.44, 7.10 |
| Owns dog [Yes vs. No] | 0.56 | -2.22, 3.35 | -3.23 ^*^ | -6.11, -0.35 | -0.65 | -3.41, 2.10 | -1.72 | -4.94, 1.49 |
| Owns car [Yes vs. No] | 1.38 | -2.14, 4.90 | -0.49 | -3.73, 2.75 | 1.39 | -1.39, 4.18 | 2.86 | -1.47, 7.18 |
| Days active [1-4 vs. 0] | 4.88 ^**^ | 1.69, 8.07 | 6.30 ^***^ | 3.28, 9.32 | 5.93 ^***^ | 2.84, 9.02 | 5.56 ^***^ | 2.54, 8.57 |
| Days active [5+ vs. 0] | 3.56 | -0.51, 7.63 | 8.56 ^***^ | 4.68, 12.44 | 7.75 ^***^ | 4.15, 11.35 | 7.50 ^***^ | 3.09, 11.91 |
| Summer | -0.16 | -3.88, 3.57 | 0.67 | -3.13, 4.47 | -0.03 | -3.31, 3.25 | 2.79 | -1.01, 6.58 |
| Autumn | -0.00 | -3.50, 3.49 | -1.80 | -5.45, 1.84 | -2.02 | -5.15, 1.12 | 2.48 | -1.29, 6.26 |
| Winter | 0.67 | -2.76, 4.11 | 0.61 | -3.00, 4.23 | 2.75 | -0.35, 5.84 | 0.90 | -2.81, 4.62 |
| Observations | 980 | | 859 | | 948 | | 965 | |
| R^2^ adj | 0.203 / 0.169 | | 0.221 / 0.181 | | 0.286 / 0.253 | | 0.211 / 0.176 | |

**Supplementary Table S5 continued***:* WHO-5 scores for each country separately

|  | **Germany** | | **Greece** | | **Hong Kong (China)** | | **Ireland** | |
| --- | --- | --- | --- | --- | --- | --- | --- | --- |
| *Predictors* | *Estimates* | *CI* | *Estimates* | *CI* | *Estimates* | *CI* | *Estimates* | *CI* |
| (Intercept) | 48.02 ^***^ | 39.78, 56.26 | 50.24 ^***^ | 42.90, 57.58 | 49.30 ^***^ | 34.08, 64.51 | 44.36 ^***^ | 36.08, 52.64 |
| Greenspace [Q2 vs. Q1] | 1.70 | -2.40, 5.80 | -1.25 | -4.77, 2.26 | -2.00 | -7.69, 3.70 | 4.20 ^*^ | 0.31, 8.08 |
| Greenspace [Q3 vs. Q1] | -0.08 | -4.03, 3.86 | 0.12 | -3.85, 4.09 | -2.49 | -8.35, 3.37 | 4.15 ^*^ | 0.16, 8.14 |
| Greenspace [Q4 vs. Q1] | 0.84 | -3.00, 4.69 | -0.62 | -4.65, 3.41 | -4.11 | -10.77, 2.54 | 3.65 ^*^ | 0.07, 7.24 |
| Inland blue [Yes vs. No] | -0.18 | -2.87, 2.51 | -2.24 | -6.88, 2.40 | 0.04 | -4.19, 4.28 | 1.49 | -1.44, 4.42 |
| Coastal blue [Yes vs. No] | -3.49 | -16.74, 9.75 | -0.37 | -3.79, 3.05 | 0.70 | -3.15, 4.54 | 5.00 ^*^ | 1.15, 8.84 |
| Green | 0.12 | -0.09, 0.32 | 0.54 ^***^ | 0.34, 0.74 | 0.11 | -0.17, 0.38 | 0.39 ^***^ | 0.20, 0.58 |
| Green ^2^ | 0.00 | -0.01, 0.01 | -0.00 | -0.01, 0.00 | 0.01 | -0.00, 0.02 | -0.00 | -0.01, 0.00 |
| Inland blue | 0.36 ^*^ | 0.03, 0.69 | -0.16 | -0.53, 0.21 | 0.53 ^*^ | 0.06, 0.99 | 0.01 | -0.28, 0.29 |
| Inland blue ^2^ | -0.01 ^*^ | -0.03, -0.00 | 0.01 | -0.01, 0.03 | -0.02 | -0.03, 0.00 | 0.00 | -0.01, 0.01 |
| Coastal blue | 0.35 | -0.07, 0.77 | 0.04 | -0.21, 0.28 | 0.06 | -0.39, 0.51 | -0.14 | -0.40, 0.13 |
| Coastal blue ^2^ | -0.01 | -0.02, 0.01 | 0.00 | -0.00, 0.01 | -0.01 | -0.03, 0.01 | 0.00 | -0.00, 0.01 |
| INS | -0.33 | -4.44, 3.78 | 4.40 ^*^ | 0.04, 8.76 | 7.61 ^**^ | 2.94, 12.29 | 3.17 | -0.41, 6.76 |
| INS^2^ | 0.19 | -0.29, 0.66 | -0.39 | -0.88, 0.11 | -0.65 ^*^ | -1.25, -0.05 | -0.18 | -0.61, 0.25 |
| Sex [Male vs. Female] | 0.36 | -2.40, 3.12 | 0.28 | -2.54, 3.11 | 0.85 | -2.41, 4.11 | 0.28 | -2.34, 2.89 |
| Age [30-39 vs. 18-29] | 1.38 | -4.09, 6.85 | 3.65 | -0.69, 7.98 | 2.86 | -1.85, 7.57 | -1.62 | -6.00, 2.76 |
| Age [40-49 vs. 18-29] | 1.53 | -3.88, 6.95 | 3.51 | -1.06, 8.09 | 6.40 ^*^ | 1.26, 11.54 | -3.11 | -7.83, 1.60 |
| Age [50-59 vs. 18-29] | 5.04 | -0.20, 10.29 | 2.85 | -1.85, 7.56 | 6.07 ^*^ | 0.38, 11.75 | 0.98 | -4.08, 6.04 |
| Age [60+ vs. 18-29] | 7.29 ^**^ | 1.89, 12.70 | 4.46 | -2.23, 11.15 | 6.75 | -0.88, 14.39 | 3.68 | -1.87, 9.22 |
| HH income [Q2 vs. Q1] | 0.27 | -4.31, 4.85 | 3.36 | -0.59, 7.30 | -6.52 | -17.91, 4.87 | 1.72 | -2.94, 6.37 |
| HH income [Q3 vs. Q1] | 3.61 | -1.26, 8.49 | 3.34 | -1.05, 7.73 | 2.19 | -9.09, 13.47 | 5.16 ^*^ | 0.47, 9.85 |
| HH income [Q4 vs. Q1] | 7.15 ^**^ | 2.14, 12.15 | 7.42 ^**^ | 2.27, 12.58 | 0.58 | -9.78, 10.95 | 5.09 | -0.06, 10.24 |
| HH income [Q5 vs. Q1] | 9.24 ^***^ | 3.78, 14.70 | 4.42 | -3.46, 12.31 | -0.81 | -10.81, 9.19 | 4.28 | -1.30, 9.86 |
| HH income [prefer not answer vs. Q1] | 2.34 | -2.37, 7.04 | 5.72 ^*^ | 0.91, 10.53 | -5.56 | -16.66, 5.54 | 4.84 | -0.19, 9.87 |
| Retired [vs. Unemployed] | 4.00 | -1.82, 9.81 | 3.20 | -3.34, 9.75 | 9.54 | -2.56, 21.64 | 5.39 | -0.22, 10.99 |
| Home maker [vs. Unemployed] | -2.27 | -11.04, 6.49 | 3.34 | -4.29, 10.97 | 6.41 | -6.17, 18.99 | 0.65 | -5.43, 6.74 |
| In education [vs. Unemployed] | 2.05 | -5.30, 9.39 | 8.47 ^*^ | 1.74, 15.19 | 2.94 | -7.15, 13.03 | 3.07 | -3.30, 9.45 |
| Employed [vs. Unemployed] | 0.72 | -4.43, 5.88 | 5.70 ^**^ | 1.97, 9.42 | 3.44 | -4.51, 11.38 | 1.87 | -2.46, 6.20 |
| University ed.[ Yes vs. No] | -1.15 | -4.35, 2.04 | 0.05 | -2.92, 3.02 | 0.02 | -3.57, 3.61 | 1.02 | -1.66, 3.69 |
| Ill/disability [Yes vs. No] | -13.95 ^***^ | -16.83, -11.06 | -10.84 ^***^ | -14.30, -7.38 | -5.69 ^***^ | -9.05, -2.32 | -9.98 ^***^ | -12.66, -7.30 |
| Married [Yes vs. No] | 0.95 | -3.33, 5.23 | -3.11 | -6.57, 0.34 | 3.44 | -0.74, 7.61 | 2.54 | -1.24, 6.32 |
| Adults in HH [2 vs. 1] | -2.28 | -6.69, 2.14 | -0.78 | -4.75, 3.18 | 1.35 | -4.84, 7.54 | -1.45 | -5.90, 3.00 |
| Adults in HH [3+ vs. 1] | 1.68 | -3.38, 6.75 | -0.28 | -4.27, 3.72 | 1.20 | -4.78, 7.17 | -1.36 | -5.78, 3.06 |
| Children in HH [1 vs. 0] | -0.49 | -5.33, 4.35 | -1.24 | -4.96, 2.47 | -0.92 | -5.22, 3.39 | -0.78 | -4.42, 2.86 |
| Children in HH[2+ vs. 0] | -4.58 | -10.09, 0.94 | -2.78 | -7.32, 1.76 | -0.86 | -6.36, 4.64 | 4.48 ^*^ | 0.36, 8.60 |
| Owns dog [Yes vs. No] | -2.02 | -5.52, 1.49 | -1.15 | -4.16, 1.85 | 0.20 | -4.92, 5.31 | 0.46 | -2.24, 3.15 |
| Owns car [Yes vs. No] | 3.88 ^*^ | 0.12, 7.63 | 4.48 | -0.34, 9.30 | 2.53 | -1.13, 6.19 | 1.77 | -2.03, 5.56 |
| Days active [1-4 vs. 0] | 7.66 ^***^ | 4.28, 11.05 | 3.79 ^*^ | 0.84, 6.74 | 2.25 | -1.54, 6.03 | 6.02 ^***^ | 2.54, 9.50 |
| Days active [5+ vs. 0] | 8.97 ^***^ | 4.72, 13.22 | 5.09 ^*^ | 0.22, 9.96 | 2.70 | -4.80, 10.19 | 9.24 ^***^ | 5.17, 13.32 |
| Summer | 2.20 | -1.76, 6.15 | 1.62 | -2.33, 5.58 | 2.26 | -2.23, 6.76 | 0.35 | -3.33, 4.02 |
| Autumn | 0.34 | -3.36, 4.05 | 0.67 | -3.21, 4.55 | -1.37 | -5.77, 3.03 | -0.25 | -3.72, 3.23 |
| Winter | 1.50 | -2.21, 5.21 | 0.79 | -3.07, 4.65 | -1.92 | -6.51, 2.67 | -0.95 | -4.39, 2.49 |
| Observations | 917 | | 815 | | 632 | | 930 | |
| R^2^ adj | 0.271 / 0.236 | | 0.245 / 0.205 | | 0.226 / 0.172 | | 0.243 / 0.208 | |

**Supplementary Table S5 continued***:* WHO-5 scores for each country separately

|  | **Italy** | | **Netherlands** | | **Portugal** | | **Spain** | |
| --- | --- | --- | --- | --- | --- | --- | --- | --- |
| *Predictors* | *Estimates* | *CI* | *Estimates* | *CI* | *Estimates* | *CI* | *Estimates* | *CI* |
| (Intercept) | 35.92 ^***^ | 26.38, 45.46 | 48.62 ^***^ | 41.85, 55.39 | 54.74 ^***^ | 43.90, 65.58 | 53.29 ^***^ | 44.78, 61.81 |
| Greenspace [Q2 vs. Q1] | 3.80 | -0.14, 7.74 | 1.07 | -2.14, 4.29 | 2.47 | -1.50, 6.43 | 0.62 | -2.73, 3.96 |
| Greenspace [Q3 vs. Q1] | 4.82 ^*^ | 1.09, 8.55 | 2.78 | -0.42, 5.99 | 3.89 | -0.13, 7.92 | 2.16 | -0.90, 5.22 |
| Greenspace [Q4 vs. Q1] | 4.54 ^*^ | 0.10, 8.98 | 1.65 | -2.18, 5.48 | 4.23 | -0.40, 8.87 | -1.72 | -5.61, 2.18 |
| Inland blue [Yes vs. No] | 1.64 | -1.72, 5.00 | -1.11 | -3.60, 1.37 | -3.81 ^*^ | -7.26, -0.36 | -2.18 | -5.29, 0.94 |
| Coastal blue [Yes vs. No] | 0.00 | -4.28, 4.29 | 0.74 | -6.62, 8.09 | 3.45 | -0.82, 7.71 | 3.14 | -0.88, 7.16 |
| Green | 0.17 | -0.01, 0.35 | 0.18 ^*^ | 0.01, 0.36 | 0.32 ^***^ | 0.14, 0.51 | 0.03 | -0.15, 0.21 |
| Green ^2^ | -0.00 | -0.01, 0.00 | 0.00 | -0.00, 0.01 | -0.00 | -0.01, 0.00 | 0.00 | -0.00, 0.01 |
| Inland blue | 0.26 | -0.03, 0.56 | 0.10 | -0.17, 0.38 | 0.06 | -0.22, 0.35 | 0.44 ^***^ | 0.19, 0.70 |
| Inland blue ^2^ | -0.01 | -0.01, 0.00 | -0.00 | -0.02, 0.01 | 0.00 | -0.01, 0.01 | -0.01 ^*^ | -0.01, -0.00 |
| Coastal blue | 0.16 | -0.09, 0.42 | 0.05 | -0.29, 0.39 | 0.27 ^*^ | 0.01, 0.52 | 0.24 ^*^ | 0.02, 0.46 |
| Coastal blue ^2^ | 0.00 | -0.01, 0.01 | 0.00 | -0.01, 0.02 | -0.00 | -0.01, 0.00 | -0.00 | -0.01, 0.00 |
| INS | 0.60 | -4.09, 5.29 | 0.44 | -3.23, 4.10 | 4.17 | -0.16, 8.49 | 3.52 | -0.35, 7.38 |
| INS^2^ | 0.22 | -0.29, 0.73 | 0.07 | -0.36, 0.50 | -0.29 | -0.77, 0.19 | -0.24 | -0.68, 0.21 |
| Sex [Male vs. Female] | 1.20 | -1.63, 4.03 | -0.86 | -3.33, 1.60 | -0.23 | -3.00, 2.54 | -0.58 | -3.02, 1.86 |
| Age [30-39 vs. 18-29] | -0.99 | -6.17, 4.19 | -0.09 | -4.61, 4.44 | -1.82 | -6.93, 3.29 | -2.16 | -6.47, 2.15 |
| Age [40-49 vs. 18-29] | 1.37 | -3.85, 6.59 | 4.12 | -0.26, 8.50 | -1.86 | -6.94, 3.23 | -3.97 | -8.32, 0.39 |
| Age [50-59 vs. 18-29] | -1.48 | -6.85, 3.88 | 6.32 ^**^ | 1.92, 10.72 | -0.32 | -5.44, 4.79 | -1.80 | -6.45, 2.84 |
| Age [60+ vs. 18-29] | 3.00 | -3.09, 9.08 | 8.86 ^***^ | 4.09, 13.64 | -1.54 | -7.76, 4.67 | 0.65 | -4.60, 5.90 |
| HH income [Q2 vs. Q1] | 1.40 | -3.41, 6.20 | -3.20 | -7.95, 1.56 | -1.14 | -6.32, 4.03 | 0.31 | -4.31, 4.94 |
| HH income [Q3 vs. Q1] | 2.52 | -2.07, 7.10 | -0.00 | -4.66, 4.66 | -3.57 | -8.42, 1.29 | 3.07 | -1.34, 7.47 |
| HH income [Q4 vs. Q1] | 3.84 | -1.01, 8.69 | 2.20 | -2.47, 6.86 | -2.11 | -6.65, 2.43 | -0.10 | -4.97, 4.77 |
| HH income [Q5 vs. Q1] | 2.99 | -2.24, 8.22 | 2.94 | -1.89, 7.76 | 3.79 | -1.19, 8.78 | 2.15 | -2.27, 6.56 |
| HH income [prefer not answer vs. Q1] | 3.21 | -2.14, 8.56 | -3.10 | -7.57, 1.37 | -1.98 | -7.20, 3.24 | -1.11 | -6.66, 4.43 |
| Retired [vs. Unemployed] | 4.94 | -1.76, 11.63 | 7.46 ^**^ | 2.70, 12.23 | 9.63 ^**^ | 3.07, 16.19 | 8.25 ^**^ | 2.34, 14.15 |
| Home maker [vs. Unemployed] | 5.05 | -1.70, 11.80 | 4.96 | -0.69, 10.61 | 5.63 | -4.13, 15.39 | 5.59 | -1.35, 12.54 |
| In education [vs. Unemployed] | 6.47 | -0.97, 13.92 | 0.76 | -5.60, 7.11 | 8.52 ^*^ | 0.89, 16.16 | -1.17 | -7.82, 5.49 |
| Employed [vs. Unemployed] | 5.40 ^*^ | 0.55, 10.25 | 6.90 ^***^ | 3.43, 10.37 | 9.24 ^***^ | 4.72, 13.75 | 6.13 ^**^ | 2.08, 10.18 |
| University ed.[ Yes vs. No] | 1.57 | -1.26, 4.41 | -1.17 | -3.67, 1.33 | -2.74 | -5.56, 0.09 | 0.34 | -2.28, 2.96 |
| Ill/disability [Yes vs. No] | -7.17 ^***^ | -10.12, -4.21 | -9.70 ^***^ | -12.24, -7.16 | -6.58 ^***^ | -9.88, -3.27 | -7.99 ^***^ | -11.04, -4.94 |
| Married [Yes vs. No] | -0.05 | -3.77, 3.67 | -0.30 | -4.21, 3.60 | 1.87 | -1.58, 5.31 | -0.25 | -3.73, 3.22 |
| Adults in HH [2 vs. 1] | -0.33 | -4.79, 4.12 | 4.83 ^*^ | 0.59, 9.07 | 0.24 | -4.23, 4.71 | -0.73 | -5.22, 3.75 |
| Adults in HH [3+ vs. 1] | -1.17 | -5.61, 3.27 | 3.82 | -0.48, 8.13 | -3.80 | -8.34, 0.75 | 1.25 | -3.16, 5.66 |
| Children in HH [1 vs. 0] | 0.07 | -3.80, 3.94 | -0.07 | -4.17, 4.03 | -1.30 | -4.91, 2.31 | -1.45 | -4.75, 1.85 |
| Children in HH[2+ vs. 0] | 2.31 | -2.00, 6.62 | -3.98 | -8.22, 0.27 | -2.58 | -7.03, 1.86 | -0.15 | -3.88, 3.59 |
| Owns dog [Yes vs. No] | 2.08 | -0.78, 4.94 | 0.95 | -2.01, 3.91 | 1.69 | -1.21, 4.59 | 0.98 | -1.52, 3.48 |
| Owns car [Yes vs. No] | 7.79 ^**^ | 2.17, 13.41 | -0.55 | -3.80, 2.70 | 0.99 | -6.31, 8.29 | 6.74 ^**^ | 1.91, 11.57 |
| Days active [1-4 vs. 0] | 6.31 ^***^ | 3.03, 9.58 | 5.54 ^***^ | 2.65, 8.44 | 2.27 | -0.75, 5.29 | 1.21 | -2.27, 4.68 |
| Days active [5+ vs. 0] | 7.70 ^**^ | 2.96, 12.44 | 7.19 ^***^ | 3.52, 10.87 | 8.15 ^***^ | 3.79, 12.51 | 3.15 | -0.87, 7.16 |
| Summer | -0.01 | -3.93, 3.90 | 1.71 | -1.59, 5.02 | 1.34 | -2.69, 5.37 | -1.15 | -4.62, 2.32 |
| Autumn | -1.62 | -5.26, 2.03 | 1.80 | -1.44, 5.03 | 0.96 | -2.89, 4.81 | 2.21 | -1.16, 5.58 |
| Winter | -2.41 | -6.03, 1.20 | 1.26 | -1.98, 4.49 | 0.86 | -3.00, 4.73 | 1.09 | -2.25, 4.44 |
| Observations | 925 | | 1003 | | 787 | | 880 | |
| R^2^ adj | 0.243 / 0.208 | | 0.237 / 0.204 | | 0.227 / 0.185 | | 0.205 / 0.166 | |

**Supplementary Table S5 continued***:* WHO-5 scores for each country separately

|  | **Sweden** | | **United Kingdom** | |
| --- | --- | --- | --- | --- |
| *Predictors* | *Estimates* | *CI* | *Estimates* | *CI* |
| (Intercept) | 49.23 *** | 42.29, 56.18 | 38.14 *** | 30.95, 45.33 |
| Greenspace [Q2 vs. Q1] | -1.57 | -4.99, 1.86 | 0.08 | -2.86, 3.01 |
| Greenspace [Q3 vs. Q1] | 0.66 | -2.85, 4.17 | 0.60 | -2.52, 3.73 |
| Greenspace [Q4 vs. Q1] | -0.68 | -4.95, 3.59 | -0.28 | -3.80, 3.24 |
| Inland blue [Yes vs. No] | 0.32 | -2.26, 2.90 | -0.51 | -2.87, 1.84 |
| Coastal blue [Yes vs. No] | -0.45 | -3.85, 2.96 | 0.70 | -3.38, 4.77 |
| Green | 0.32 *** | 0.14, 0.50 | 0.18 | -0.01, 0.36 |
| Green ^2^ | -0.01 * | -0.01, -0.00 | -0.00 | -0.01, 0.00 |
| Inland blue | -0.10 | -0.42, 0.22 | -0.04 | -0.38, 0.31 |
| Inland blue ^2^ | 0.01 | -0.01, 0.03 | 0.01 | -0.01, 0.03 |
| Coastal blue | 0.46 ** | 0.17, 0.76 | 0.14 | -0.14, 0.43 |
| Coastal blue ^2^ | -0.01 | -0.02, 0.00 | -0.00 | -0.01, 0.01 |
| INS | 0.66 | -3.01, 4.32 | 3.59 * | 0.26, 6.91 |
| INS^2^ | -0.02 | -0.44, 0.41 | -0.26 | -0.67, 0.14 |
| Sex [Male vs. Female] | 2.68 * | 0.20, 5.16 | 2.60 * | 0.27, 4.94 |
| Age [30-39 vs. 18-29] | -1.78 | -5.99, 2.44 | -0.89 | -5.59, 3.82 |
| Age [40-49 vs. 18-29] | 1.98 | -2.35, 6.30 | 1.40 | -3.12, 5.93 |
| Age [50-59 vs. 18-29] | 5.11 * | 0.60, 9.61 | 4.07 | -0.48, 8.61 |
| Age [60+ vs. 18-29] | 12.29 *** | 7.00, 17.57 | 8.20 *** | 3.33, 13.08 |
| HH income [Q2 vs. Q1] | 1.61 | -3.32, 6.54 | 2.46 | -1.60, 6.51 |
| HH income [Q3 vs. Q1] | 0.84 | -4.30, 5.98 | 0.59 | -3.59, 4.77 |
| HH income [Q4 vs. Q1] | 0.65 | -4.40, 5.70 | 3.31 | -1.09, 7.71 |
| HH income [Q5 vs. Q1] | 3.74 | -1.33, 8.82 | 3.62 | -0.84, 8.08 |
| HH income [prefer not answer vs. Q1] | -1.80 | -6.85, 3.24 | 5.77 ** | 1.92, 9.62 |
| Retired [vs. Unemployed] | 0.61 | -4.89, 6.11 | 8.35 *** | 3.62, 13.08 |
| Home maker [vs. Unemployed] | -2.50 | -11.12, 6.11 | 4.83 | -1.19, 10.85 |
| In education [vs. Unemployed] | 9.25 *** | 3.79, 14.71 | 10.38 ** | 3.97, 16.79 |
| Employed [vs. Unemployed] | 2.40 | -1.25, 6.05 | 7.27 *** | 3.22, 11.31 |
| University ed.[ Yes vs. No] | -0.29 | -2.83, 2.24 | 1.34 | -1.03, 3.70 |
| Ill/disability [Yes vs. No] | -12.89 *** | -15.44, -10.35 | -10.76 *** | -13.17, -8.34 |
| Married [Yes vs. No] | 2.64 | -1.54, 6.82 | 3.13 | -0.32, 6.58 |
| Adults in HH [2 vs. 1] | -1.87 | -6.33, 2.59 | -0.05 | -3.89, 3.80 |
| Adults in HH [3+ vs. 1] | -0.59 | -5.42, 4.24 | 1.93 | -2.03, 5.90 |
| Children in HH [1 vs. 0] | 0.67 | -3.27, 4.61 | -2.87 | -6.79, 1.05 |
| Children in HH[2+ vs. 0] | 0.64 | -3.36, 4.65 | 0.66 | -3.57, 4.88 |
| Owns dog [Yes vs. No] | -2.35 | -5.63, 0.92 | -4.55 ** | -7.27, -1.83 |
| Owns car [Yes vs. No] | 5.34 ** | 2.09, 8.59 | 2.30 | -0.93, 5.53 |
| Days active [1-4 vs. 0] | 3.93 * | 0.71, 7.15 | 4.94 *** | 2.19, 7.70 |
| Days active [5+ vs. 0] | 4.28 * | 0.50, 8.06 | 8.05 *** | 4.61, 11.50 |
| Summer | -0.79 | -4.43, 2.85 | 0.75 | -2.76, 4.26 |
| Autumn | 2.66 | -0.64, 5.95 | -1.23 | -4.56, 2.10 |
| Winter | -1.60 | -4.92, 1.72 | -2.42 | -5.70, 0.85 |
| Observations | 930 | | 1116 | |
| R^2^ adj | 0.286 / 0.253 | | 0.269 / 0.241 | |

Q = quartile for greenspace and quintile for income; HH = Household; INS = Inclusion of Nature in Self Scale; Married includes cohabiting; Seasons are approximate since ‘Spring’ data were collected in June and referred to the ‘last 4 weeks’ (i.e. May-June), ‘Summer’ in September (i.e. August-September), ‘Autumn’ in December (November to December), and ‘Winter’ in March (i.e. February – March), seasons were reversed for Australia; *^*^ p<0.05  ^**^ p<0.01   ^***^ p<0.001.*

**Supplementary Figure S1**: Country-level variance (i.e. intercept variance) across the four mental health outcomes.


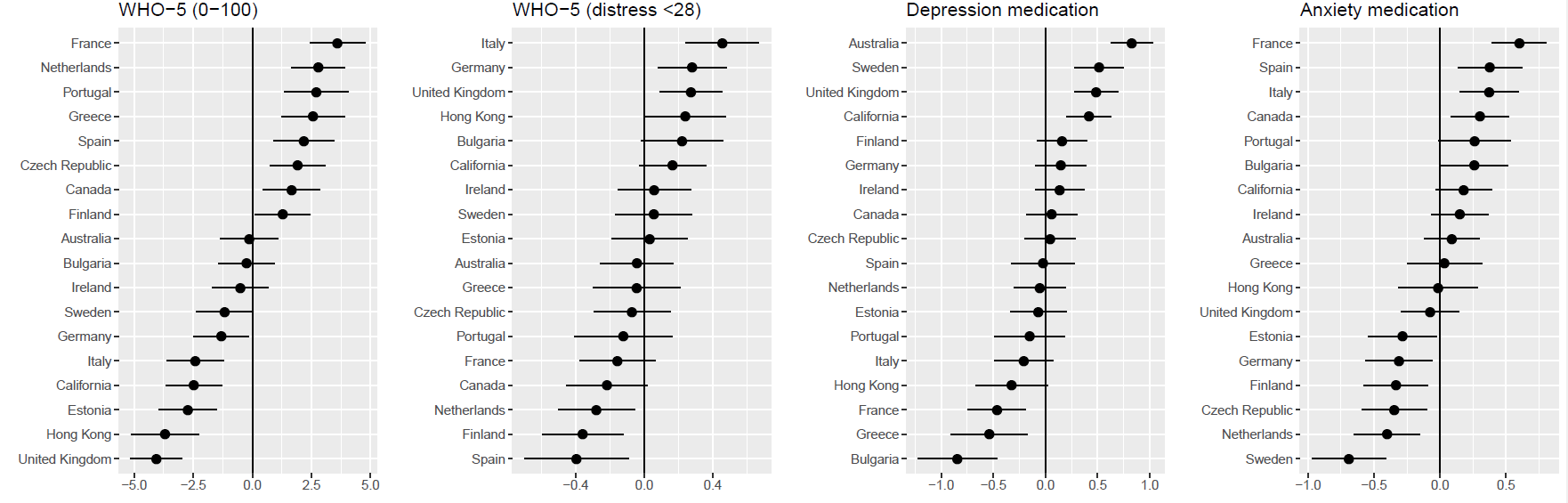

Supplement: Supplementary file 1 — Supplementary Information. [file 41598_2021_87675_MOESM1_ESM.docx]
